# Naphthalene Exchange in [Re(η^6^‐napht)_2_]^+^ with Pharmaceuticals Leads to Highly Functionalized Sandwich Complexes [M(η^6^‐pharm)_2_]^+^ (M=Re/^99m^Tc)

**DOI:** 10.1002/chem.202103566

**Published:** 2021-12-13

**Authors:** Qaisar Nadeem, Federica Battistin, Olivier Blacque, Roger Alberto

**Affiliations:** ^1^ Department of Chemistry University of Zurich Winterthurerstr. 190 8057 Zurich Switzerland

**Keywords:** bioorganometallics, rhenium, sandwich complexes, technetium, theranostics

## Abstract

Bis‐arene sandwich complexes are generally prepared by the Fischer‐Hafner reaction, which conditions are incompatible with most O‐ and N‐ functional groups. We report a new way for the synthesis of sandwich type complexes [Re(η^6^‐arene)_2_]^+^ and [Re(η^6^‐arene)(η^6^‐benzene)]^+^ from [Re(η^6^‐napht)_2_]^+^ and [Re(η^6^‐napht)(η^6^‐benzene)]^+^, with functionalized arenes and pharmaceuticals. N‐methylpyrrolidine (NMP) facilitates the substitution of naphthalene with the incoming arene. A series of fully characterized rhenium sandwich complexes with simple arenes, such as aniline, as well as with active compounds like lidocaine and melatonin are presented. With these rhenium compounds in hand, the radioactive sandwich complexes [^99m^Tc(η^6^‐pharm)_2_]^+^ (pharm=pharmaceutical) can be unambiguously confirmed. The direct labelling of pharmaceuticals with ^99m^Tc through η^6^‐coordination to phenyl rings and the confirmation of the structures with the rhenium homologues opens a path into molecular theranostics.

## Graphical abstract

**To overcome** the limits of the Fischer‐Hafner reaction, [Re(η^6^‐napht)_2_]^+^ and [Re(η^6^‐napht)(benzene)]^+^ have been used to prepare sandwich complexes [Re(η^6^‐arene)_2_]^+^ and [Re(η^6^‐arene)(η^6^‐benzene)]^+^. NMP is used to promote the substitution of naphthalene with simple arenes as well as with active compounds like lidocaine and melatonin. This compound can be used to unambiguously confirm the radioactive sandwich complexes [^99m^Tc(η^6^‐pharm)_2_]^+^.

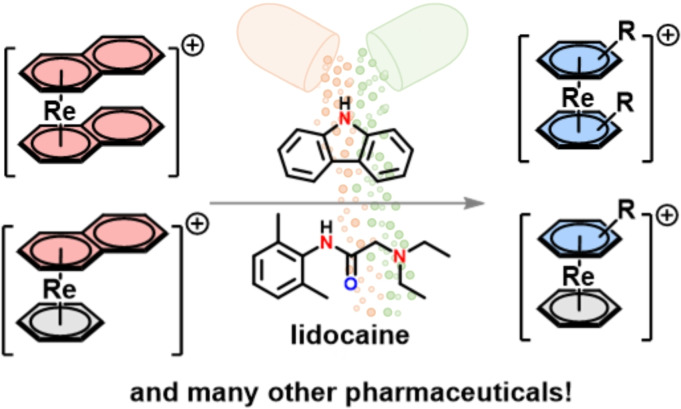

## Introduction

**Scheme 1:**
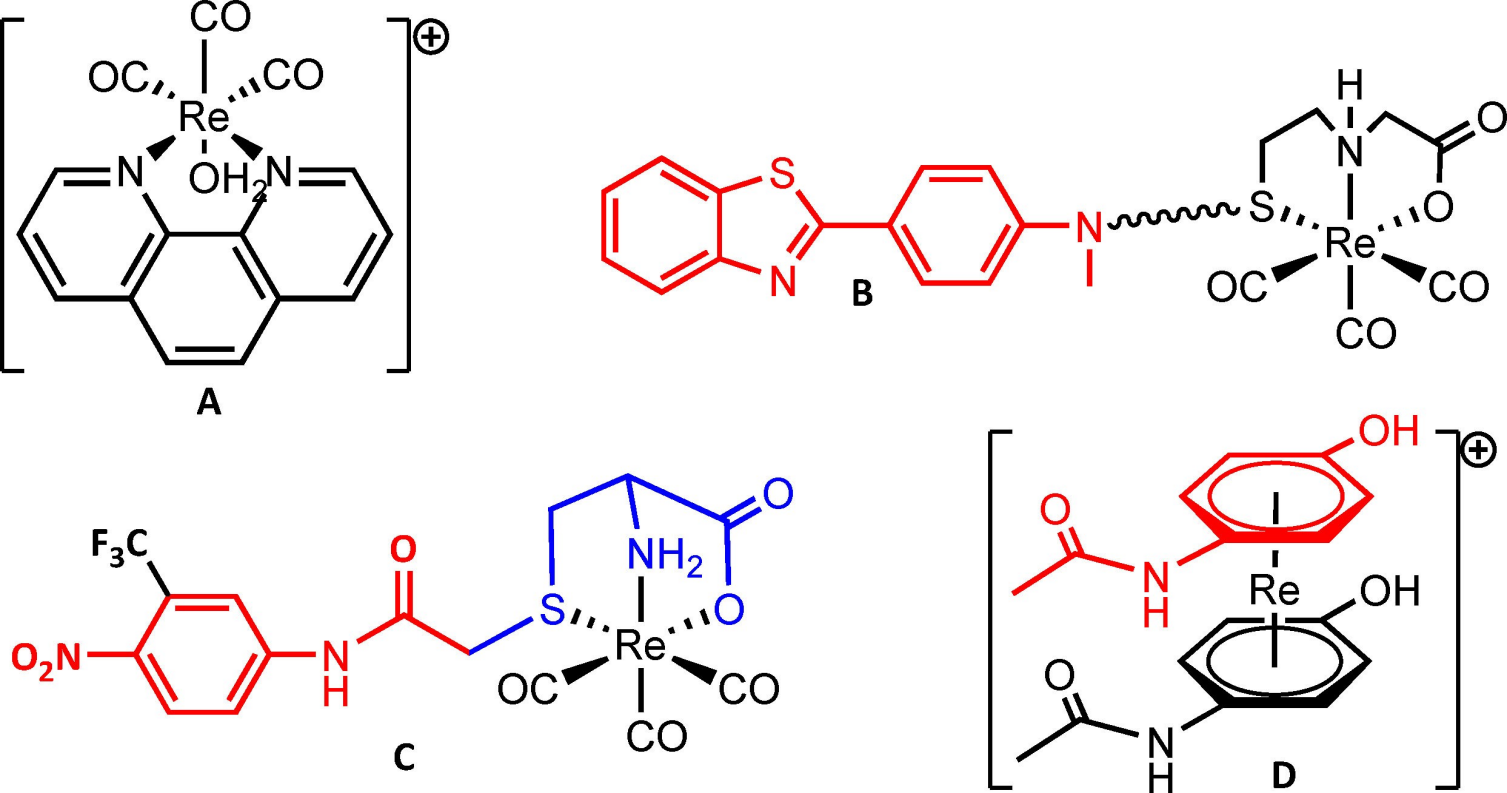
Concepts: A) De novo bioactive metal complex,^[3b]^ B) complex tagged to bioactive moiety in red,^[8]^ C) pharmacophore in which a substructure is replaced with a complex (blue),^[9]^ and D) a complex in which the lead structure remains unchanged.^[10]^

Bioorganometallic complexes of rhenium and other transition elements have gained a lot of momentum over the past years, complementing the available armory of inorganic medicinal compounds.^[1]^ Conceptually, these complexes can be divided in i) “de novo” compounds with structures of no immediately obvious biological function, or ii) tagged compounds where the overall structure of the lead is kept intact and the complex is bound to ligands conjugated to the lead.^[2]^ Alternatively and more recently, a substructure of a pharmaceutically active compound is replaced by the metal complex (Scheme 1). Examples for these concepts comprise the recent isocyanide complexes of Wilson and coworkers (TRIP), which proved to be therapeutically very active.^[3]^ The famous ferrocifen and its derivatives by Jaouen et al.^[4]^ or the light‐induced activation of complexes presented by Gasser et al. exemplify the alternative concepts.^[5]^ The “tagged concept” is common for the preparation of molecules in molecular imaging with ^99m^Tc but is not frequently applied with non‐radioactive compounds. Compounds with no structural changes in the active lead structures are rare since coordination sites in biologically active pharmacophores are often not available or not appropriately arranged for metal coordination. Exceptions are here curcumins, which offer an acetylacetonato‐like coordination site.^[6]^ A further example is the [Cr°(CO)_3_] complex of estradiol or amino acids in which the chromium is directly coordinated to the phenyl ring in the organic structure.^[7]^

Challenged by the question of introducing rhenium or ^99m^Tc in a pharmaceutically active structure with minimal alteration of their functional groups (D in Scheme 1), we recently came up with the concept of directly coordinating the two elements to arenes, ubiquitous in drugs. To follow the same strategy as with the above‐mentioned chromium complex but with [M(CO)_3_]^+^ (M=Re/^99m^Tc) instead, is tempting but not an option since the corresponding [Re(η^6^‐C_6_H_6_)(CO)_3_]^+^ complexes are labile and loose the arene ring rapidly.^[11]^

Earlier, we reported about the preparation of [Re(η^6^‐C_6_H_6_)_2_]^+^ and long‐lived [^99^Tc(η^6^‐C_6_H_6_)_2_]^+^ along the classical Fischer‐Hafner approach, that is, using Zn/AlCl_3_ as activation/reduction agents, and adapting an improved preparation by Kudinov et al.^[12]^ The stabilities of these sandwiches against water and air turned out to be extraordinarily high, which makes these moieties very suitable for application in bioorganometallic chemistry. In a follow up study, we showed that complexes [^99m^Tc(η^6^‐arene)_2_]^+^ with arene being functionalized phenyls, are accessible in water in one step and directly from [^99m^TcO_4_]^−^.^[10]^ This approach offers a general approach to directly labelled pharmaceuticals according to structure D in Scheme 1. It is thus not necessary to introduce additional chelators. Thereby, the functional groups and one face of the pharmaceutical are left unchanged.

Obviously, Fischer‐Hafner conditions are not applicable for functionalized arenes such as found in pharmaceuticals. The reactions with ^99m^Tc in water are only possible due to the high dilution of the metal. We were thus in the peculiar situation of being able to make the organometallic ^99m^Tc compounds but not the corresponding rhenium homologues. Aiming at molecular theranostics with homologous compounds of rhenium for therapy and ^99m^Tc for imaging, a more flexible way had to be found for the former element. We now present in this study an alternative approach towards [Re(η^6^‐pharm)_2_]^+^(pharm=mequinol, lidocaine and others) starting from [Re(η^6^‐napht)_2_]^+^ (**1** ^+^, napht=naphthalene) and catalyzed by N‐methyl‐pyrrolidone (NMP) in low to medium yields. We also present the preparation of the corresponding ^99m^Tc homologues. The rhenium and the ^99m^Tc matched‐pairs offer thus a generally applicable path towards the direct labelling of pharmaceuticals through η^6^‐coordination to phenyl rings, integrated in the lead. This opens a path into molecular theranostics.

## Results and Discussion

### Rhenium sandwich complexes

For the preparation of functionalized [Re(η^6^‐arene)_2_]^+^ complexes, Fischer‐Hafner conditions are not appropriate since AlCl_3_ is not compatible with most functional O‐ or N‐groups. So far, the sole approach was the substitution of ligands in preformed Re^I^ complexes. The mutual exchange of coordinated arenes in sandwich complexes by other incoming ligands, for example other arenes, is quite common for group 8 or group 9 elements iron and ruthenium, iridium or rhodium, but essentially unknown for those of group 7.^[13]^ Since the basic complex [Re(η^6^‐C_6_H_6_)_2_]^+^ is extremely stable, any attempts to replace one or both benzene ligands failed under any conditions. This contrasts reactions with for example the corresponding Ru^2+^ compounds.^[13b]^ Due to the lack of other suitable Re^I^ starting materials, we attempted to substitute naphthalene in [Re(η^6^‐napht)_2_]^+^ since naphthalene is generally weaker bound than benzene due to the more extended π‐system.

**Scheme 2:**
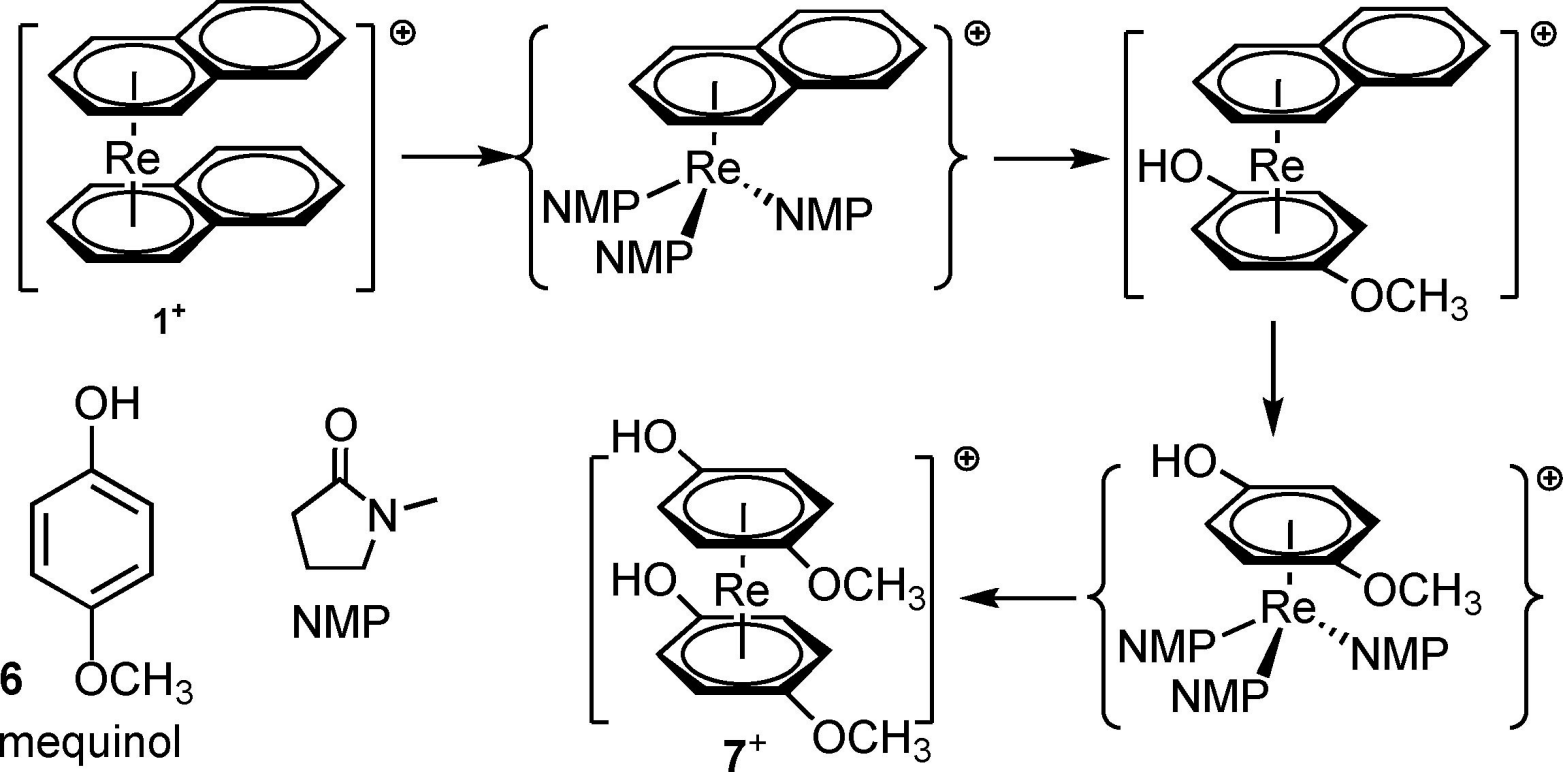
Possible mechanism path B for the stepwise substitution of naphthalene in [Re(η^6^‐naphth)_2_]^+^ with mequinol in dioxane, catalyzed by NMP.

**Scheme 3:**
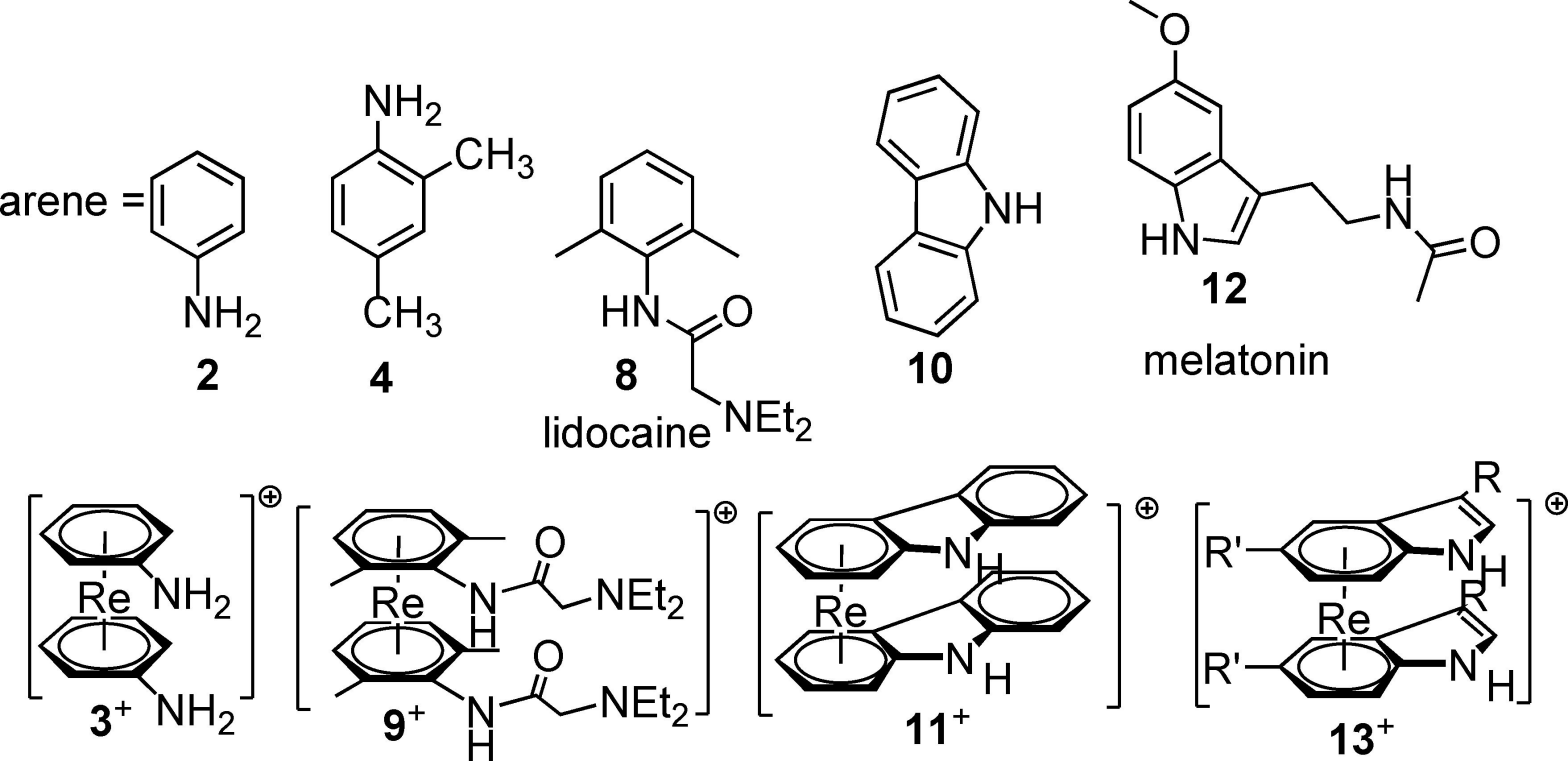
A series of functionalized arenes and pharmaceuticals (top row) and their corresponding rhenium sandwich complexes (bottom row) prepared by stepwise substitution of naphthalene in **1** ^+^. Typical reaction conditions: 25 equiv. incoming arene, 5 equiv. NMP in 1,4‐dioxane at 120–130 °C.

For substituting naphthalene in **1** ^+^, there are two principal options: either using the incoming ligand directly as solvent (path A) or choosing an appropriate solvent mixture (path B). For the latter strategy, mechanistically, the naphthalene should be first replaced by weakly coordinating solvents to yield tentative intermediates [Re(η^6^‐napht)(sol)_3_]^+^. In these, the incoming arene substitutes the solvent ligands to yield the products. A second and equal step will replace the 2^nd^ naphthalene to achieve the complex [Re(η^6^‐pharm)_2_]^+^ (Scheme 2). The approach A only makes sense if the incoming arene ligand is liquid and/or not an expensive compound, which is rarely the case for pharmaceuticals. Since benzene and derivatives are much more stably bound than naphthalene, the reverse reaction is not expected to occur. We investigated many solvents as stabilizing ligands for a Re^I^ intermediate with a once cleaved naphthalene. It turned out that N‐methylpyrrolidine (NMP) was the only one that gave satisfying yields (or yields at all) with a broader variety of arenes. A number of ligands/pharmaceuticals that have been investigated along this line are shown together with the respective complexes in Scheme 3.

**Figure 1:**
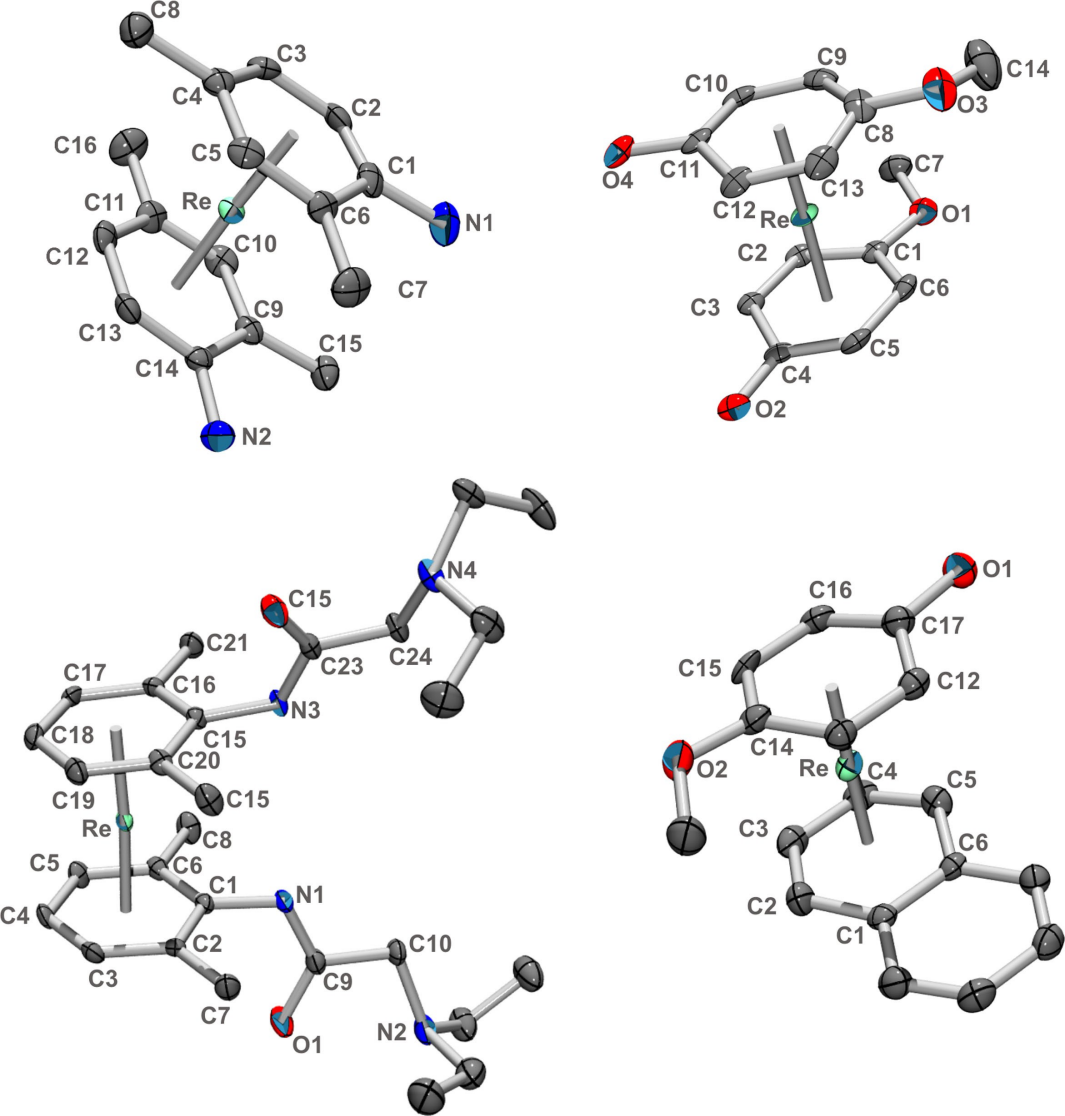
Ellipsoid displacement plots of complexes [Re(η^6^‐**4**)_2_]^+^ (**5** ^+^, 2,4‐dimethylaniline, one isomer), [Re(η^6^‐**6**)_2_]^+^(**7** ^+^, mequinol), [Re(η^6^‐**8**)_2_]^+^(**9** ^+^, lidocaine) and [Re(η^6^‐**6**)(η^6^‐napht)]^+^ (**14** ^+^). Counter‐ions and hydrogens are omitted for clarity. X‐ray crystallographic details can be found in the Supporting Information. All ellipsoids are drawn at 20 % probability.

For aniline **2** and 2,4‐dimethylaniline **4**, path A has been exemplified before by us but isolation from excess ligand and purification were demanding.^[10]^ The reaction with ligand **4** as solvent gave [Re(η^6^‐**4**)_2_]^+^ (**5** ^+^) and its X‐ray structure could now be solved and is shown in Figure 1 for completion.^[10]^ For comparison and to corroborate the options of path B, aniline (**2**) and **1** ^+^ were directly reacted in dioxane as solvent in the presence of 5 equiv. of NMP to obtain [Re(η^6^‐**2**)_2_]^+^(**3** ^+^) in 39 % yield after purification (all the yields are calculated after purification with prep‐HPLC). The reaction without NMP did not give any yield at all. Mequinol (**6**, 4‐methoxyphenol), a simple phenyl with two substituents, is a drug used in skin depigmentation (Scheme 2).^[14]^ The respective sandwich complexes with Re and ^99m^Tc are not intended for application, but they are further examples for sandwich complexes with two (four) reactive substituents, which cannot be prepared by Fischer‐Hafner methods. The reaction with mequinol also followed procedure B. Mequinol (50 equiv.) and complex **1** ^+^ were dissolved in dioxane and the solution was heated to 130 °C for 86 h. Compound [Re(η^6^‐**6**)_2_]^+^ (**7** ^+^) was thus obtained in 18 % yield (for all experimental details and characterizations see Supporting Information). Lidocaine is a local anesthetic and an even higher functionalized arene. The reaction with **8** (lidocaine) was performed analogously to obtain the corresponding rhenium complex [Re(η^6^‐**8**)_2_]^+^ (**9** ^+^) in 4 % yield after purification. To proceed from single arenes to fused ring system, the indole‐based carbazole system is a prototypical ligand since carbazole subunits are present in many pharmaceuticals. Three anti‐cancer drugs based on carbazole structures, Celiptium®, Alecensa®, Rydapt® are already in the market for cancer therapy.^[15]^ The preparation of [Re(η^6^‐**10**)_2_]^+^ (**11** ^+^) with carbazole was done differently. This complex was synthesized by dropwise addition of a solution of **1** ^+^ in NMP over 15 h into a solution of carbazole in dioxane at 120 °C, resulting in two diastereomers (see below) in a collective yield of about 31 %. Melatonin (**12**), an endogenous hormone regulating the wake‐sleep cycle, is well known to many of us. It comprises the indole subunit and is a derivatized extension of it. As for **11** ^+^, a solution of **1** ^+^ in NMP was added dropwise to a NMP solution of **12** over 10 h. The complex [Re(η^6^‐**12**)_2_]^+^ (**13** ^+^) was isolated in 34 % yield in the form of its two diastereomers (see below). The different synthetic strategies vary mainly due to solubility reasons. Since carbazole and melatonin are only slightly soluble in dioxane, NMP was taken in excess. Experimental details for all syntheses and compound characterizations can be found in the Supporting Information.

In all the reactions, progress can be monitored by UPLC/MS. The intermediates with coordinated NMP could not be detected, probably due to reactivity with water/acetonitrile from the mobile phase. We could clearly detect, however, the mono‐substituted compounds still comprising one naphthalene. For the reaction with mequinol **6**, this intermediate [Re(η^6^‐**6**)(η^6^‐napht)]^+^(**14** ^+^) could be isolated and was structurally characterized (see Figure 1).

The yields for the bis‐substituted sandwich complexes are generally low, mainly due to side reactions and formation of [ReO_4_]^−^ over the long reaction times. During the reactions, we detected by UPLC‐MS in most cases reduced **1** ^+^, in which one of the coordinated naphthalenes was reduced to dihydro‐naphthalene. Similarly, we detected mono‐substituted side‐products in which the remaining naphthalene was also reduced to dihydro‐naphthalene. In the synthesis with carbazole to complex **11** ^+^ for example, [Re(η^6^‐**10**)(η^6^‐dihydronapht)]^+^ and further reduced to [Re(η^6^‐**10**)(η^6^‐tetraline)]^+^ were observed along the reaction by UPLC‐MS. Since these corresponding sandwich complexes are essentially benzene complexes, they do not exchange the dihydronaphthalene or the tetraline anymore. The reductive side reactions are one of the main reasons why the yields are low. We emphasize at this point again that any attempt to substitute benzene by any means instead of naphthalene was unsuccessful. Benzene or other aromatic hydrocarbons are extremely stably bound. The X‐ray structures of complexes [**5**](ReO_4_), [**7**](tfa), [**9**](ReO_4_), and [**14**](PF_6_) could be elucidated and are shown in Figure 1 and the Supporting Information respectively.

The structures do not show unexpected features. They all have in common that the binding centroid is shifted with respect to the ring centroid by 0.056, 0.062, 0.016, 0.025, 0.083, 0.063 Å, respectively for the complexes of aniline, 2,4‐dimethylaniline, mequinol, lidocaine, carbazole (η^6^‐**U**)_2_ and carbazole (η^6^‐**U**)(η^6^‐**D**) (see below). This is most distinct for the complexes with an amino group directly bound to the arene due to the delocalization of the N lone pair into the aryl ring.

### Stereochemical features

**Scheme 4:**
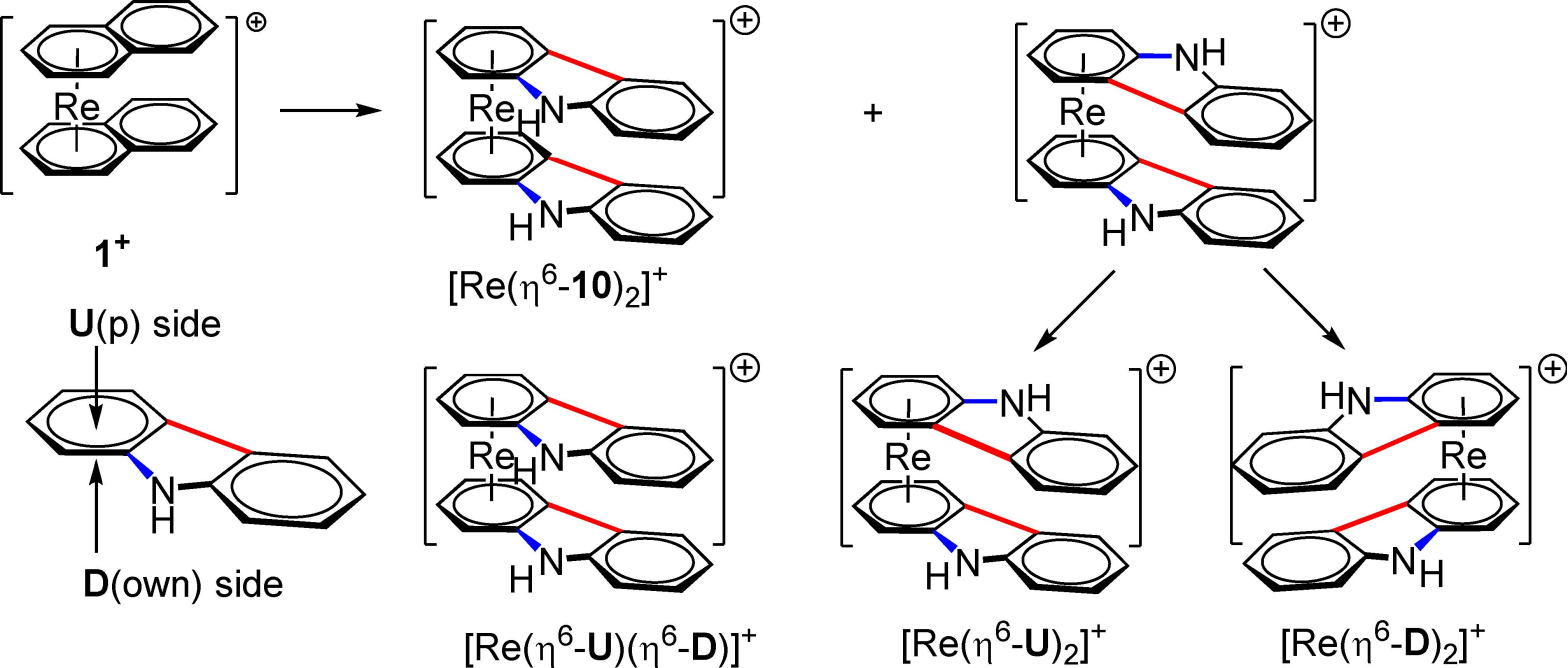
Substitution of naphthalene with carbazole giving **11** ^+^. Coordination from the two different arene‐planes promotes planar chirality; one meso‐form [Re(η^6^‐**U**)(η^6^‐**D**)]^+^ and one pair of enantiomers [Re(η^6^‐**U**)_2_]^+^ and [Re(η^6^‐**D**)_2_]^+^ is obtained.

**Figure 2:**
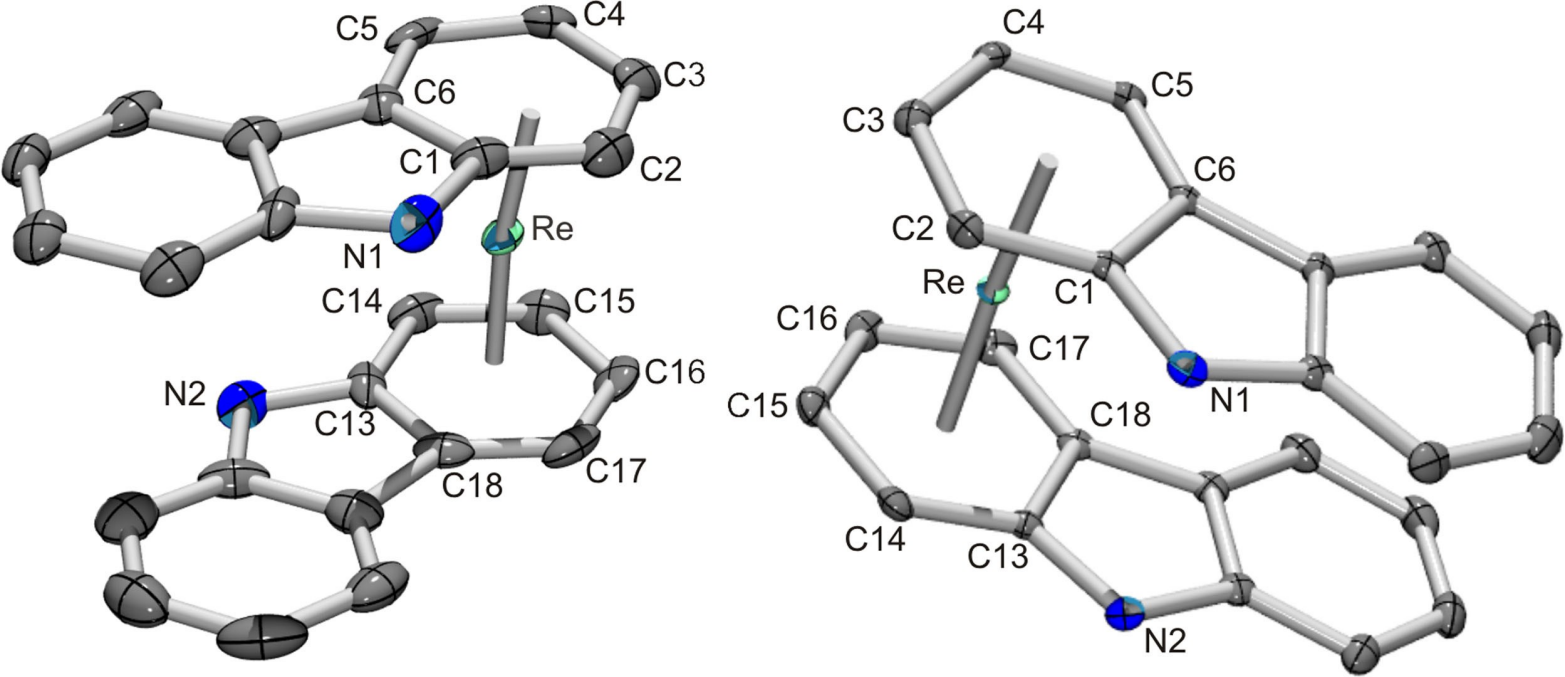
Ellipsoid displacement plot of one of the enantiomers of **11** ^+^ representing the [Re(η^6^‐**U**)_2_]^+^ form (left) and the meso‐form [Re(η^6^‐**U**)(η^6^‐**D**)]^+^ (right) as shown in Scheme 4. Ellipsoids are drawn at 20 % probability.

**Figure 3:**
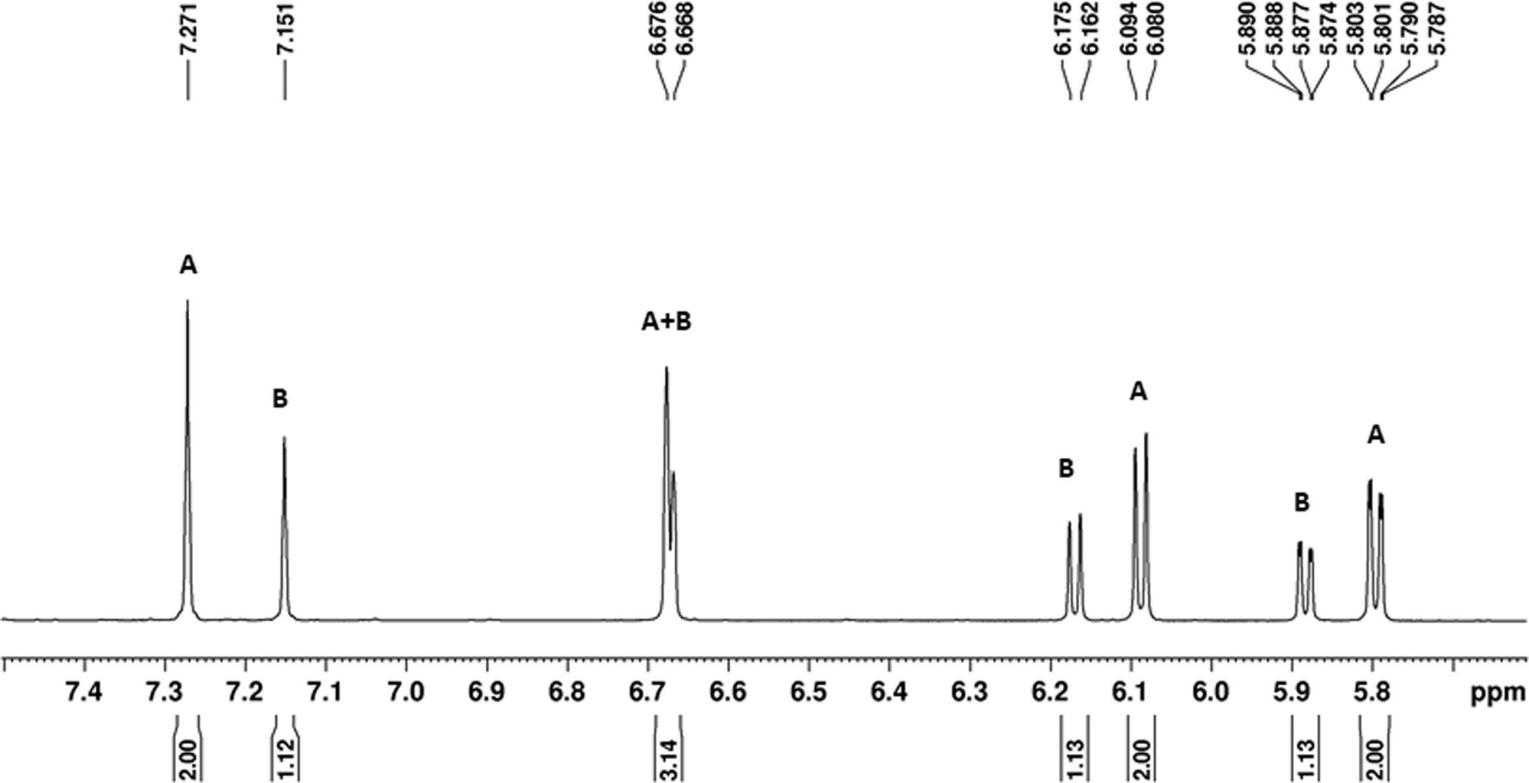
^1^H NMR spectrum of [Re(η^6^‐melat)_2_]^+^ **13** ^+^ in the aromatic region, evidencing the two diastereomers A and B (the meso‐form and the enantiomeric pair).

Depending on the configuration of the incoming arenes, some interesting stereochemical features become evident. Multi‐substituted arenes such as 2,4‐dimethylaniline (**4**), carbazole (**10**) or melatonin (**12**) can coordinate from both sides, from **U**p and from **D**own (Scheme 4) of the arene. If the arene has no mirror‐plane perpendicular to the ring as in **4** or two different substituents as in **10** and **12**, the option of coordinating from two different sides leads to the planar chirality phenomenon in the final complexes. Such planar chirality features are mainly found in ferrocene or Ru‐sandwich complexes but are very rare for rhenium.^[16]^ If the arenes coordinate from the two opposite planes **U** and **D**, the resulting sandwich complex [Re(η^6^‐**U**)(η^6^‐**D**)]^+^ will be in a meso‐form. If coordination occurs from the same sides either U or D, the two enantiomers [Re(η^6^‐**U**)_2_]^+^and [Re(η^6^‐**D**)_2_]^+^ will be obtained as shown in Scheme 4. If there are no or minor steric constraints, there is no preference for either side and the two diastereomers are obtained in about equal yields. The ellipsoid displacement plot of [Re(η^6^‐**4**)_2_]^+^ in Figure 1 represents [Re(η^6^‐**U**)_2_]^+^ but its enantiomer is also present in the crystal since the space group is P2_1_/c. If the diastereomers are chemically different enough, they can be distinguished by HPLC. Occasionally, they can even be separated from the reaction, as it is the case for the carbazole sandwiches shown in Scheme 4. The enantiomers of [**11**]BF_4_ could be separated from the meso‐form and be crystallized. Both enantiomers are of course present in the crystal. An ellipsoid displacement plot of one of them is given in Figure 2. Generally, the diastereomers are discernable in their NMR spectra (Figure 3). Not shown here but the same stereochemical features are also occurring when a chirality center is present in a single side chain of the arene. With such an enantiomerically pure arene, two diastereomers will be found as well, a meso‐form and one pair of enantiomers. For biological applications, the presence of different stereoisomers may be a concern. It offers on the other hand the option of exploring the interaction of stereochemical features and bioactivities.

Obviously, the same stereochemical considerations apply also to reactions with ^99m^Tc (see below). Two discernable peaks are sometimes found in the radio‐HPLC analyses, which can eventually be separated from each other by “preparative” radio‐HPLC. In case of rhenium, ^1^H NMR analyses display two distinguishable sets of signals. Figure 3 shows an excerpt of the ^1^H NMR of the melatonin complex **13** ^+^ in which the two sets of signals in the aromatic region are nicely separated. The two diastereomers could be isolated from each other and are shown together with the full spectrum in the Supporting Information.

### Mixed‐arene rhenium sandwich complexes

**Scheme 5:**
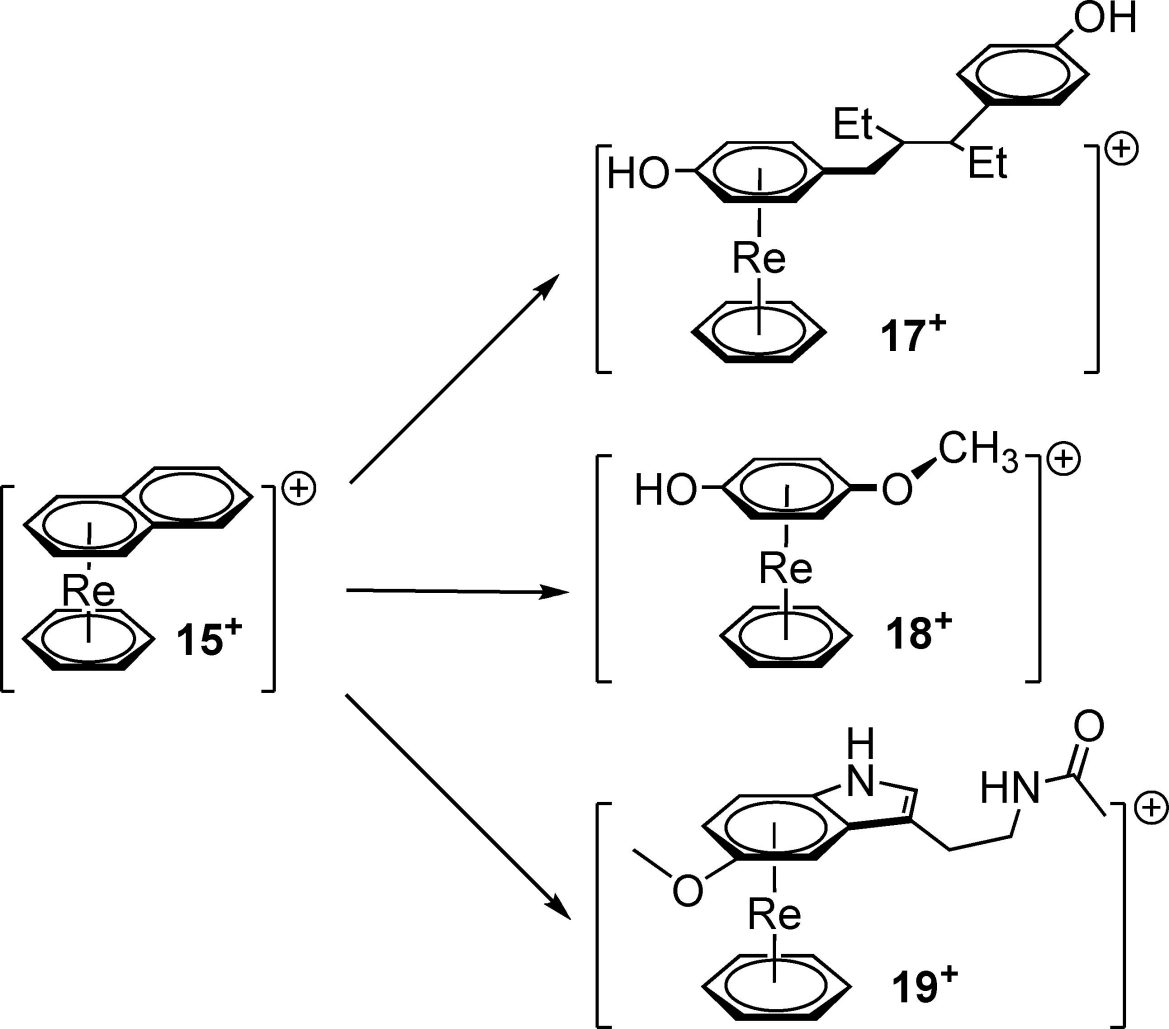
Reactions of the mixed‐arene sandwich complex **15** ^+^ with hexestrol (top) and mequinol (middle) and melatonin (bottom). Typical reaction conditions: 12.5 equiv. incoming arene, 2.5 equiv. NMP in 1,4‐dioxane at 120 °C.

The sandwich complexes described so far carry two identical and potentially bioactive ligands. For comparative reasons, it was interesting to also prepare the mono‐substituted sandwich complexes, that is, one face occupied by the functionalized arene, the other face by for example benzene or another simple arene. Given the high stability of the η^6^‐coordinated benzene, it is unlikely that it is replaced. Thus, a reaction of [Re(η^6^‐C_6_H_6_)(η^6^‐napht)]^+^ (**15** ^+^) with a pharmacophore should yield the corresponding mixed‐arene sandwich complexes [Re(η^6^‐C_6_H_6_)(η^6^‐pharm)]^+^. The preparation of the corresponding starting material **15** ^+^ was reported by us earlier.^[17]^ Its reaction with hexestrol (**16**), a compound that was previously taken for the treatment of certain hormone‐dependent cancers,^[18]^ gave in a clean reaction the mixed‐arene sandwich complex [Re(η^6^‐C_6_H_6_)(η^6^‐**16**)]^+^ (**17** ^+^). The same procedure with mequinol and with melatonin yielded the corresponding complex [Re(η^6^‐C_6_H_6_)(η^6^‐**6**)]^+^ (**18** ^+^) and [Re(η^6^‐C_6_H_6_)(η^6^‐**12**)]^+^ (**19** ^+^) (Scheme 5).

**Figure 4:**
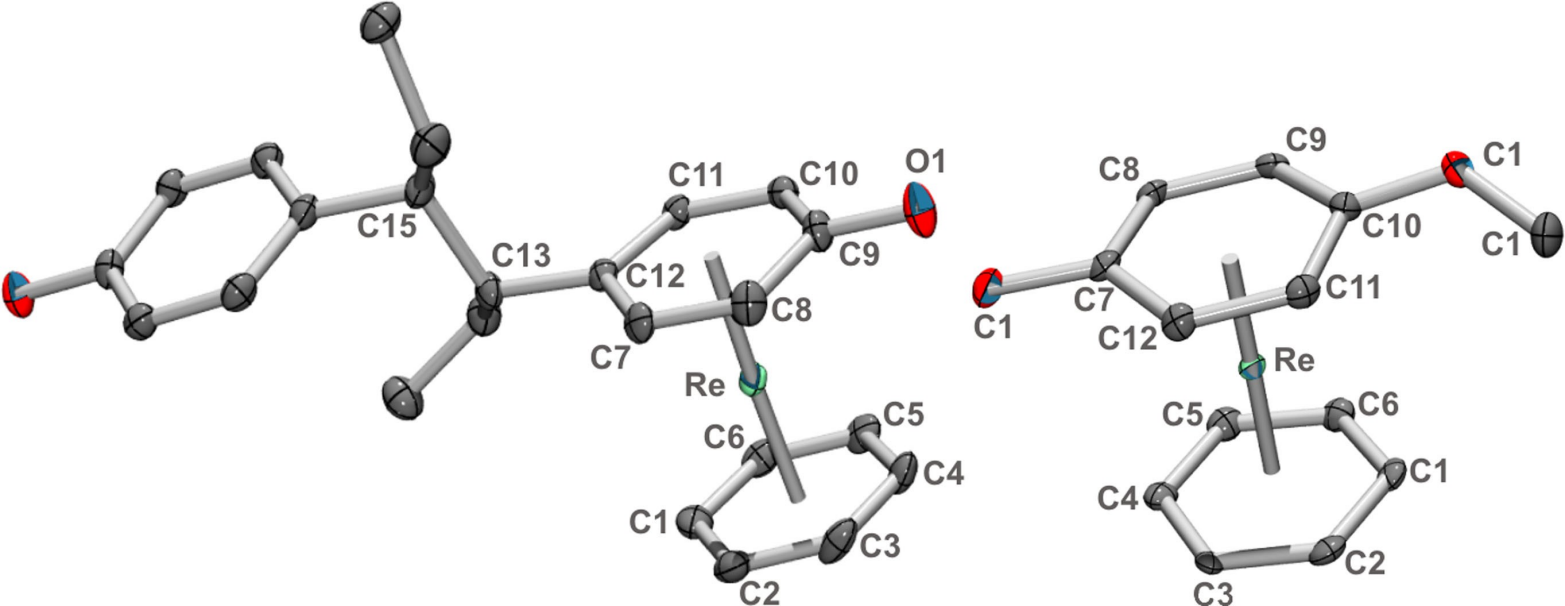
Ellipsoid displacement plots of the hexestrol complex [Re(η^6^‐C_6_H_6_)(η^6^‐**16**)]^+^ (**17** ^+^) and the mequinol complex [Re(η^6^‐C_6_H_6_)(η^6^‐**6**)]^+^ (**18** ^+^). Ellipsoids are drawn at 20 % probability.

The structures of **17** ^+^ and **18** ^+^ could be elucidated. Ellipsoid displacement plots are shown in Figure 4 and details are given in the Supporting Information.

As before, some stereochemical peculiarities may appear, depending on the nature of the incoming arene. The hexestrol used for the reaction is in its meso form. Depending on which arene binds the rhenium centre (the one closest to the *R* or *S* carbon), two enantiomers are formed. In fact, the space group of the crystal structure is P2_1_/n and both enantiomers are thus present in the crystal. Complex **19** ^+^ is formed as a mixture of two enantiomers as well, since melatonin can bind the metal from both sides.

### ^99m^Tc sandwich complexes

Apart from potential applications in bioorganometallic chemistry, the purpose for the preparation of these rhenium sandwich complexes is to have a fully characterized complex for the assessment of the authenticity of the respective ^99m^Tc homologue. HPLC retention time comparison Re/^99m^Tc is the only and FDA accepted option for this identification since concentrations of ^99m^Tc are far too low to allow for classical analytical techniques. As reported earlier by our group, the preparation of many supposed ^99m^Tc sandwich complexes is straight. For most of these new complexes, an unambiguous confirmation of their authenticities is lacking though due to the impossibility of preparing the rhenium homologues. The synthetic approaches described above allow now to prepare the respective rhenium sandwiches with higher functionalized arenes and to compare them chromatographically with their ^99m^Tc congeners.

**Scheme 6:**
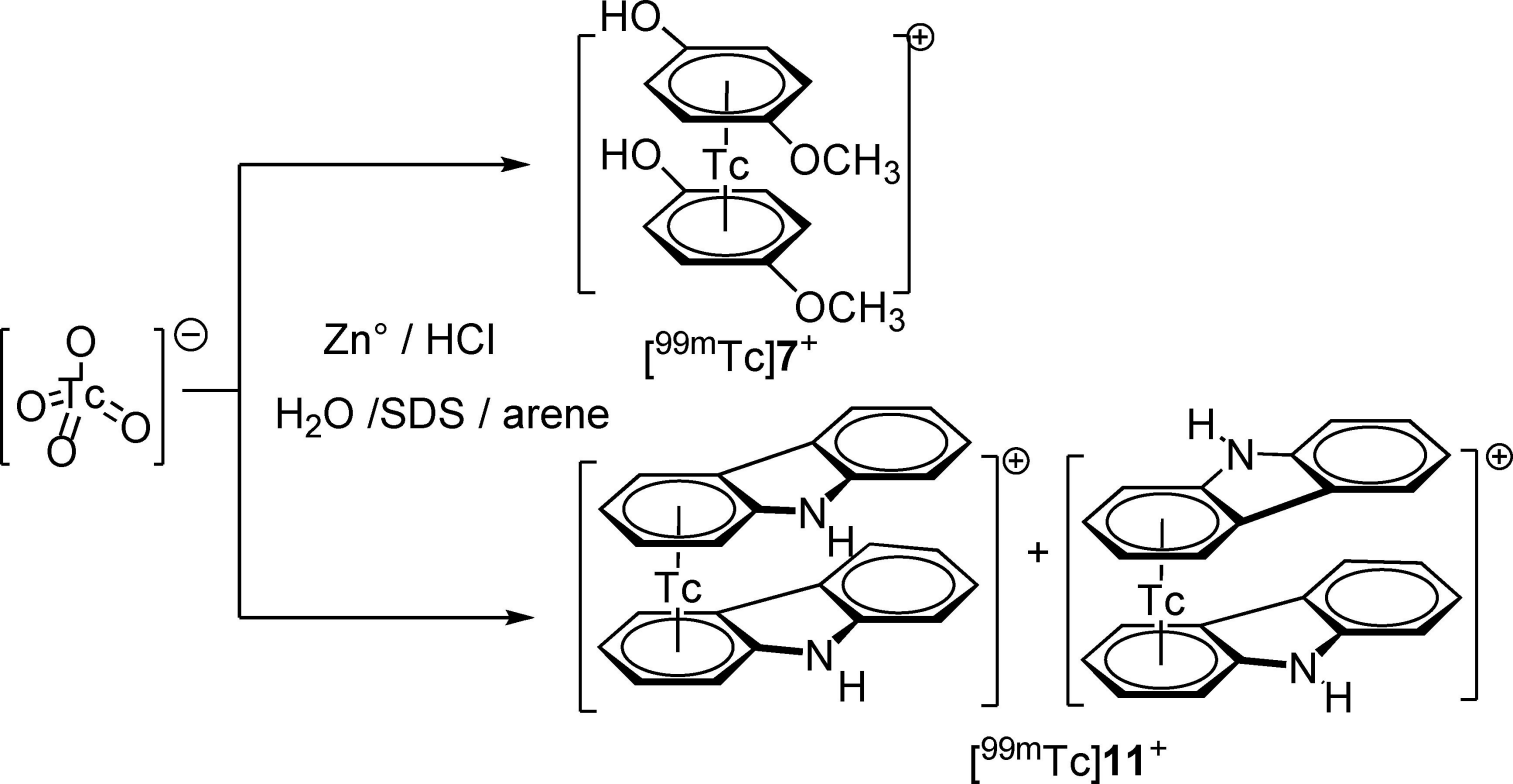
Preparation of ^99m^Tc sandwich complexes [^99m^Tc]**7^+^** and [^99m^Tc]**11^+^** directly from [^99m^TcO_4_]^−^ in water.

As shown in Scheme 6, [^99m^Tc] pertechnetate as eluted from a ^99^Mo/^99m^Tc generator is mixed with the arene of interest in water and additional ingredients as described in the Supporting Information. With Zn° as reducing agent and some acid, the sandwich complexes form as exclusive products. Details for the preparation are given in the Supporting Information. Yields of these reactions are variable, but the uncommon preparation of binary or ternary technetium sandwich complexes of the type [^99m^Tc(η^6^‐arene)_2_]^+^ and [^99m^Tc(η^6^‐arene1)(η^6^‐arene2)]^+^ directly from [^99m^TcO_4_]^−^ in water is feasible (Scheme 6).^[10]^ The concentration of ^99m^Tc is in the nanomolar range, the formation of side products is unlikely and the only complexes that are formed are of the [^99m^Tc(η^6^‐arene)_2_]^+^‐type. The reaction conditions are obviously non‐Fischer‐Hafner like. We emphasize that complexes of the type [^99m^Tc(η^6^‐arene)_2_]^+^ are extremely stable and do not decompose if exposed to air or water at any pH value.

**Figure 5:**
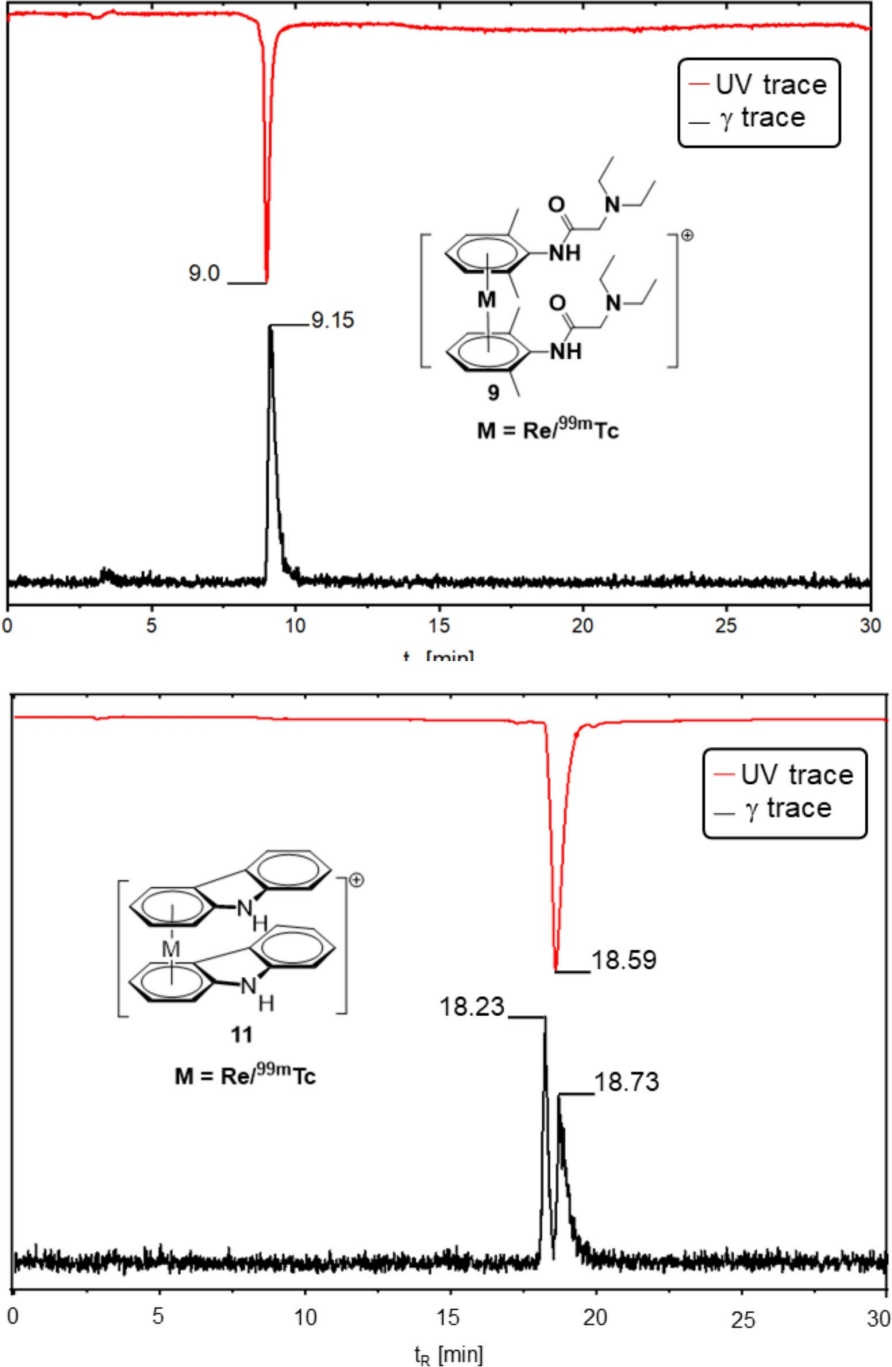
Co‐injection of **9** ^+^ (lidocaine) together with the homologue [^99m^Tc]**9** ^+^ and a trace for one of the carbazole complexes **11** ^+^ and [^99m^Tc]**11** ^+^, yielding both stereoisomers.

Despite getting one peak in the HPLC for sandwich complexes with ^99m^Tc, only comparison with rhenium unambiguously assesses the structure. The synthetic approaches reported herein allow us now to synthesize bis‐arene sandwich complexes of multiple functionalized arenes, and to compare their retention times with the ones obtained from the aqueous synthesis with ^99m^Tc. Figure 5 (top) exemplifies a co‐injection of **9** ^+^ (rhenium‐lidocaine) together with the homologue [^99m^Tc]**9** ^+^ and a trace for the carbazole complex **11** ^+^ and [^99m^Tc]**11** ^+^, displaying clearly the two diastereomers as discussed before (bottom). With this, the path to a wide variety of novel radiopharmaceuticals is paved since the compounds with ^99m^Tc can now be characterized with the fully characterized rhenium homologues.

## Conclusion

In conclusion, we report a synthetic pathway towards sandwich complexes [Re(η^6^‐arene)_2_]^+^ and [Re(η^6^‐arene)(η^6^‐benzene)]^+^ from [Re(η^6^‐napht)_2_]^+^ and [Re(η^6^‐napht)(η^6^‐benzene)]^+^. “Arene” refers to benzene derivatives or active pharmaceuticals such as anesthetics or anticancer drugs. NMP facilitates the substitution of the naphthalenes with incoming arenes under thermal heating. Our previous approach of reacting [Re(η^6^‐napht)_2_]^+^ in neat arenes under harsh conditions is thus extended to solid and highly functionalized arenes, representing pharmaceuticals. Sandwich complexes with pharmaceuticals open the option of investigating them with respect to their bioactivities. The available ^99m^Tc homologues complement therapy with concomitant imaging, enabling molecular theranostics. To confirm a supposed ^99m^Tc structure with the respective rhenium homologue is the scope of this report and this important base for any attempted theranostic approach has been accomplished. Biological studies with selected compounds are ongoing.

## Experimental Section

All experimental details and ^99m^Tc labelling‘s are described in the Supporting Information. Deposition Number(s) 2113179 (for **5**), 2113182 (for **7**), 2113180 (for **9**), 2113178 (for **11a**), 2113177 (for **11b**), 2113183 (for **14**), 2113184 (for **17**), 2113181 (for **18**) contain(s) the supplementary crystallographic data for this paper. These data are provided free of charge by the joint Cambridge Crystallographic Data Centre and Fachinformationszentrum Karlsruhe Access Structures service.

## Conflict of interest

The authors declare no conflict of interest.

## Supporting information

As a service to our authors and readers, this journal provides supporting information supplied by the authors. Such materials are peer reviewed and may be re‐organized for online delivery, but are not copy‐edited or typeset. Technical support issues arising from supporting information (other than missing files) should be addressed to the authors.

Supporting Information
