# Supplementary material for: Naphthalene Exchange in [Re(η6‐napht)2]+ with Pharmaceuticals Leads to Highly Functionalized Sandwich Complexes [M(η6‐pharm)2]+ (M=Re/99mTc)
Source: Chemistry. 2021 Dec 13;28(5):e202103566. doi: 10.1002/chem.202103566 (PMC9300139; doi:10.1002/chem.202103566)
Supplement: Supplementary file 1 — Supporting Information [file CHEM-28-0-s001.pdf]

Chemistry–A European Journal

Supporting Information

Naphthalene Exchange in $[Re(\eta^6\text{-napht})_2]^+$ with Pharmaceuticals Leads to Highly Functionalized Sandwich Complexes $[M(\eta^6\text{-pharm})_2]^+$ ($M = Re/^{99m}Tc$)

Qaisar Nadeem, Federica Battistin, Olivier Blacque, and Roger Alberto*

Table of Contents

Material and methods 3

Synthesis of Re complexes 6

General procedure for the syntheses of [$^{99m}Tc(\eta^6\text{-arene})_2]^+$ complexes 10

NMR spectra 11

^{99m}Tc labelling 22

X-ray crystallography 26

References 30

Materials and Techniques: Unless otherwise stated, all chemicals were of reagent grade or higher, obtained from commercial sources and used without further purification. Solvents for reactions were of p.a. grade or distilled prior to their use; H_2O was bi-distilled. Deuterated NMR-solvents were purchased from Armar Chemicals or Cambridge Isotope Laboratories, Inc. (UK). Reactions were carried out using standard Schlenk techniques in oven-dried (150°C) glass equipment and monitored for completion by analyzing a small sample (after suitable workup) by, UPLC or UPLC-ESI-MS. Evaporation of the solvents in vacuo was done with the rotary evaporator. Microwave assisted reactions were carried out in a Biotage Initiator microwave.

Radiochemistry: ^{99m}Tc is a radioactive isotope. All operations dealing with ^{99m}Tc must becarried out in a licensed and appropriately equipped laboratory, including state of the art radiation protection measures.

^{1}H-and^{13}C-NMR spectra: Bruker DRX 600 MHz, Bruker DRX 500 MHz, *BrukerAV2-400* (400 MHz) ; in deuterated solvents at 300 K; chemical shifts (δ) in ppm relative to residual solvent resonances (acetone-d_4 ^{1}H: δ 2.05, ^{13}C: δ 29.84; CD_3OD ^{1}H: δ 3.31, ^{13}C: δ 49.00; CD_3CN ^{1}H: δ 1.94, ^{13}C: δ 1.32); coupling constants (J) in Hz.

HR-ESI-MS: *QExactive* (*Thermo Fisher Scientific*, Bremen, Germany) equipped with a heated ESI source connected to a *Dionex Ultimate 3000* UPLC system. Samples dissolved in MeOH, MeOH/CH_2Cl_2 3:1, MeOH/H_2O 1:1, DMSO/H_2O 1:10, or H_2O at ca. 50µg mL^{-1}; injection of 1µL on-flow with an XRS auto-sampler (*CTC*, Zwingen, Switzerland)(mobile phase: MeOH + 0.1% HCOOH or CH_3CN/H_2O (2:8) + 0.1% HCOOH; flow rate 120µL mL^{-1}); ion source parameters: spray voltage 3.0 kV, capillary temperature 280°C, sheath gas 30L min^{-1}, s-lens RF level 55.0; aux gas temperature 250°C; full scan MS in alternating (+)/(−)-ESI mode; mass ranges 80–1’200, 133–2’000, or 200–3’000 amu; resolution (full width half-maximum) 70’000; automatic gain control(AGC) target 3.00 10^{6}; maximum allowed ion transfer time(IT) 30ms; mass calibration < 2 ppm accuracy for *m/z* 130.06619–1621.96509 in (+)-ESI with *Pierce*® ESI calibration solutions (*Thermo Fisher Scientific*, Rockford, USA); lock masses: ubiquitous erucamide (*m/z*338.34174,(+)-ESI).

UPLC-ESI-MS: *Waters Acquity* UPLC System coupled to a *Bruker Daltonics HCT*™ESI-MS, using an *Acquity UPLC BEH* C18 1.7µm (2.1 x 50mm) column. UPLC solvents were formic acid (0.1% in millipore water) (solvent A) and acetonitrile UPLC grade (solvent B). Applied UPLC gradient: 0–0.5min: 95% A, 5% B; 0.5–4.0min: linear gradient from 95% A, 5% B to 0% A, 100% B; 4.0–5.0min: 0% A, 100% B. The flow rate was 0.6mL min^{-1}. Detection was performed at 250 and 480nm (DAD).

Analytical UPLC: *VWR HITACHI Chromaster Ultra*system, using an *Acquity UPLC BEH*C18 1.7µm(2.1x50mm) column. UPLC solvents were trifluoroacetic acid (0.1% in millipore water) (solvent A) and acetonitrile UPLC grade (solvent B). Applied UPLC gradient: 0–0.5min: 95% A, 5% B; 0.5–4min: linear gradient from 95% A, 5% B to 0% A, 100% B; 4–5min: 0% A, 100% B. The flow rate was 0.5mL min^{-1}. Detection was performed at 250 and 480nm (DAD).

X-ray diffraction: Single-crystal X-ray diffraction data were collected at 160(1) K on a *Rigaku OD SuperNova/Atlas* area-detector (**3**, **7**, **9**, **17**, **18**) or on a *Rigaku OD XtaLAB Synergy, Dualflex, Pilatus* 200K (**5**, **11**(η^6-**U**)$_2$, **11**(η^6-**U**)(η^6-**D**), **14**) diffractometer using a single wavelength X-ray source (Cu K_α radiation: λ= 1.54184Å) from a micro-focus sealed X-ray tube and an Oxford liquid-nitrogen Cryo stream cooler. The selected suitable single crystal was mounted using polybutene oil on a flexible loop fixed on a goniometer head and immediately transferred to the diffractometer.

Pre-experiment, data collection, data reduction and analytical absorption correction[1] were performed with the program suite *CrysAlisPro*.[2] Using *Olex2*,[3] the structure was solved with the SHELXT[4] small molecule structure solution program and refined with the *SHELXL2018/3* program package[5] by full-matrix least-squares minimization on F^2. *PLATON*[6] was used to check the result of the X-ray analysis. CCDC 2113177 – 2113184 contain the supplementary crystallographic data for this paper. These data are provided free of charge by The Cambridge Crystallographic Data Centre *via* www.ccdc.cam.ac.uk/structures.

Radioactive materials: Na[^{99m}TcO$_4$] in 0.9% saline was eluted from a ^{99}Mo/^{99m}Tc *Ultratechnekow® FM* generator purchased from *b.e. imaging AG* (Switzerland).

HPLC analyses of ^{99m}Tc complexes: *Merck Hitachi Chromaster 5160* pump coupled to a *Merck Hitachi Chromaster 5430* diode array detector and a radiodetector. UV-vis detection was performed at 250nm. The detection of radioactive ^{99m}Tc complexes was performed with a *Berthold FlowStar LB 514* radiodetector equipped with a *BGO-X* cell. Separations were achieved on a *Macherey-Nagel NUCLEOSIL®*C18 5µm, 100 Å (250 × 3mm) column. HPLC solvents were trifluoroacetic acid (0.1% in Millipore water) (solvent A) and HPLC grade acetonitrile (solvent B). Applied HPLC gradient for purification of [^{99m}Tc(mequinol)$_2$]$^+$, [^{99m}Tc(lidocaine)$_2$]$^+$ and all co-injections [^{99m}Tc(pharmaceutical)$_2$]$^+$ (**G1**): 0-3min: 95% A (5% B); 3-3.1min: 95% A (5% B) to 75% A (25% B); 3.1-9min: 75% A (25% B); 9-9.1min: 75% A (25% B) to 66% A (34% B); 9.1-20min: 66% A (34% B) to 0% A (100% B); 20-25min: 0% A (100% B); 25-25.1min: 0% A (100% B) to 95% A (5%B); 25.1-30min: 95% A (5% B).

HPLC gradient for purification of [^{99m}Tc(carbazole)$_2$]$^+$ (**G2**): 0.1% TFA in H_2O (solvent A) and acetonitrile (solvent B), 0-5min: 95% A (5% B); 5-35min: 95% A (5% B) to 30% A (70% B); 35-40min: 30% A (70% B) to 0% A (100% B).
HPLC gradient for purification of [^{99m}Tc(melatonin)$_2$]$^+$ (**G3**): 0.1% TFA in H_2O (solvent A) and acetonitrile (solvent B), 0-5min: 95% A (5% B); 5-40min: 95% A (5% B) to 0% A (100% B).

Preparative HPLC: Shimadzu eco LC-20AP system, using a *Dr. Maisch Reprosil* C18 100-7 (40 x250mm) column for complexes **3**, **7**, **11**, **13**, **14**, **17**, **18**, **19** and LaPrep Sigma HPLC system (Knauer/ VWR) equipped with a UV detector, a sample collector and a Nucleosil 100-7 C18 250/21 column (Macherey–Nagel) for **9**. HPLC solvents were trifluoroacetic acid (0.1%in Millipore water) (solvent A) and HPLC grade acetonitrile (solvent B), with flow rate 40mL/min.
Gradient for [Re(η^6-mequinol)$_2$]$^+$ (7$^+$) and [Re(η^6-mequinol)(η^6-naphthalene)]$^+$(14$^+$) (G4): detection at 260nm, 0-5min: 75% A (25% B); 5-40min: linear gradient from 75% A (25% B) to 60% A (40% B); 40-45min: 60% A (40% B) to 0% A (100% B); 45-55min: 100% B.

Gradient for Re(η^6-lidocaine)$_2$]$^+$(9$^+$) (G5): Detection at 277nm, 0-10min: 95% A (5% B); 10-45 min: linear gradient from 95% A (5% B) to 85% A (15% B); 45-55min: 85% A (15% B) to 60% A (40% B); 55-60min: from 60% A (40% B) to 0% A (100% B); 60-70min: 0% A (100% B).

Gradient forRe(η^6-carbazole)$_2$]$^+$(11$^+$) (G6): Detection at 310nm, 0-5min: 80% A (20% B); 5-45min: linear gradient from 80% A (20% B) to 50% A (50% B); 45-50min: 50% A (50% B); 50-52min: from 50% A (50% B) to 0% A (100% B).

Gradient for Re(η^6-melatonin)$_2$]$^+$ (13$^+$)(G7): Detection at 300nm, 0-10min: 95% A (5% B); 10-40min: linear gradient from 95% A (5% B) to 70% A (30% B); 40-50min: 70% A (30% B); 50-70min: from 70% A (30% B) to 60% A (40% B); 70-90min: 60% A (40% B).

Gradient for [Re(η^6-benzene)(η^6-hexestrol)]$^+$ (17$^+$) (G8): detection at 270nm, 0-5min: 80% A (20% B); 5-35min: linear gradient from 80% A (20% B) to 0% A (100% B); 35-40min:100% B.

Gradient for [Re(η^6-benzene)(η^6-mequinol)]$^+$ (18^+) (G9): detection at 270nm, 0-5min: 90% A (10% B); 5-35min: linear gradient from 90% A (10% B) to 60% A (40% B); 35-40min: 60% A (40% B) to 0% A (100% B); 40-45min: 100% B.

Gradient for [Re(η^6-benzene)(η^6-melatonin)]$^+$ (19^+) (G10): detection at 280nm, 0-5min: 80% A (20% B); 5-35min: linear gradient from 80% A (20% B) to 60% A (40% B); 35-40min: 60% A (40% B) to 0% A (100% B); 40-45min: 100% B.

List of Abbreviations: ESI = electrospray ionization; Et_2O = diethyl ether; HPLC = high-performance liquid chromatography; HR = high-resolution; MS = mass spectrometry; napht = naphthalene; NMP = N-methyl-2-pyrrolidone; NMR = nuclear magnetic resonance; RCY = Radiochemical Yield; TFA = trifluoroacetic acid.

Synthesis of Re complexes:

Synthesis of [Re(η^6-aniline)$_2$]TFA (3)TFA: (**1**)PF_6 (30mg, 0.051mmol, 1eq.) was mixed with aniline (122.4µl 2.55mmol, 25eq.), dry NMP (24.6µL, 0.26 mmol, 5eq.) and dry 1,4-dioxane (0.5mL) in a Schlenk tube and heated at 130°C under N_2 for 8h. The solvent was removed under vacuum, the black residue was washed with Et_2O (3x2.5mL), purified by preparative HPLC/**G4** gradient and freeze-dried to afford (**3**)TFA (10 mg, 39%) as a pale yellow solid. The spectral data were in accordance with those reported in the literature.[7]

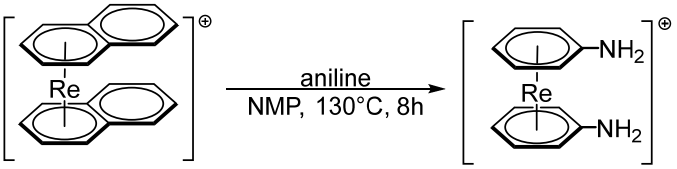

1$^+$ **3$^+$**

Scheme S1. Synthesis of [Re(η^6-aniline)$_2$]$^+$ (**3$^+$**).

Synthesis of [Re(η^6-mequinol)$_2$]TFA (7)TFA: (**1**)PF_6 (30mg, 0.051mmol, 1eq.) was mixed with mequinol (316mg, 2.55mmol, 50eq.), dry NMP (49.20µL, 0.51mmol, 10eq.) and dry 1,4-dioxane (0.5mL) in a Schlenk tube, and heated at 130°C under N_2 for 86h. The solvent was removed under vacuum and the black residue was washed with Et_2O (3x2.5mL), purified by preparative HPLC/**G4** gradient and freeze-dried to afford (**7**)TFA (5mg, 18%) as a pale yellow solid. Single crystals, suitable for X-ray diffraction analysis, were obtained by slow evaporation of a MeOH solution. ^{1}H NMR (400 MHz, CD_3OD) δ 6.05 (d, *J* = 5.5 Hz, 4H), 5.89 (d, *J* = 5.5 Hz), 3.58 (s, 6H). ^{13}C NMR (100 MHz, CD_3OD) δ 64.1, 63.5, 57.8. HRMS (ESI$^+$) *m/z* calcd. for $C_{14}H_{16}O_4Re$ [M]$^+$: 435.0601, found: 435.0595.

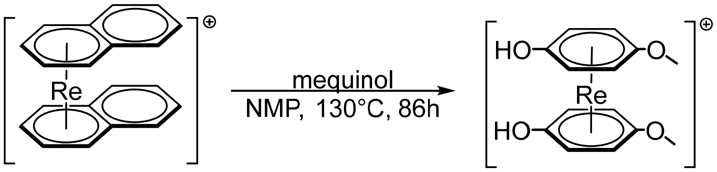

1$^+$ **7$^+$**

Scheme S2. Synthesis of [Re(η^6-mequinol)$_2$]$^+$ (**7$^+$**).

Synthesis of [Re(η^6-lidocaine)$_2$]TFA (9)TFA: (**1**)PF_6 (60mg, 0.1mmol, 1eq.) was mixed with lidocaine (586.60mg, 2.5mmol, 25eq.), dry 1,4-dioxane (0.8mL), dry NMP (48.2µL, 0.5mmol, 5eq.) in a Schlenk tube, and heated at 130°C under N_2 for 7h. The solvent was removed under vacuum; the brown residue was purified by preparative-HPLC/**G5** and freeze-dried to afford (**9**)TFA (3.4mg, 4%) as a yellow solid. Single crystals, suitable for X-ray diffraction analysis, were obtained by vapour diffusion from Et_2O (anti-solvent) into CH_3CN (solvent). ^{1}H NMR (600 MHz, CD_3CN) δ 10.82 (s, 2H), 6.03 (d, *J* = 5.1 Hz, 4H), 5.68 (t, *J* = 5.0 Hz, 2H), 4.29 (s, 4H), 3.28 (d, *J* = 6.7 Hz, 8H), 2.15 (s, 12H), 1.29 (t, *J* = 7.1 Hz, 12H). ^{13}C NMR (150 MHz, CD_3CN) δ 166.7, 99.8, 93.9, 80.7, 77.9, 53.9, 50.4, 18.1, 9.4. HRMS (ESI+) m/z calcd. for $C_{28}H_{44}N_4O_2Re$ [M]$^+$: 655.3016, found: 655.3023.

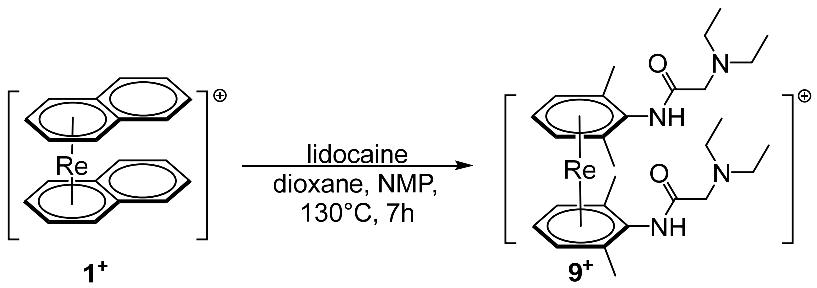

Scheme S3. Synthesis of [Re(η^6-lidocaine)$_2$]$^+$ (**9$^+$**).

Synthesis of [Re(η^6-carbazole)$_2$]TFA (11)(TFA): Solution A: (**1**)PF$_6$ (30mg, 0.051mmol, 1eq.) was dissolved in dry NMP (176μL). The red solution was put in a glass syringe (250μL, Hamilton), which was attached to an automatic pump. Solution B: Two neck Schlenk tube was charged with carbazole (213mg, 1.28mmol, 25.4eq.) and dry 1,4-dioxane (1mL) under N_2 and heated to 120°C, to form clear solution. Then solution A was added dropwise with flow rate of 17μL/h. The addition of solution A was completed in 15h; the reaction was further heated for other 7h under N_2 at 120°C. The solvent was removed under vacuum and the brown crude was purified by preparative-HPLC/**G6**, obtaining two fractions: 1 (diastereomer (η^6-**U**)$_2$, and some impurities) and 2 that is pure **11**(η^6-**U**)(η^6-**D**). After the addition of NH_4BF_4 (10mg, 0.115mmol), the product (**11**)BF$_4$ was isolated as a pale yellow solid: 5.2 mg (17% yield) for fraction **11**(η^6-**U**)$_2$ and 4.7 mg (15% yield) for fraction **11**(η^6-**U**)(η^6-**D**). Single crystals of **11**(η^6-**U**)$_2$, suitable for X-ray diffraction analysis, were obtained by vapor diffusion from Et_2O (anti solvent) into methanol (solvent). Single crystals of **11**(η^6-**U**)(η^6-**D**), suitable for X-ray diffraction analysis, were obtained by vapor diffusion from Et_2O (anti solvent) into acetone (solvent) at 5°C. ^{1}H NMR (400 MHz, CD_3OD) (diastereomer **11**(η^6-**U**)$_2$): δ 7.35 (t, *J* = 7.6 Hz, 2H), 7.27 (d, *J* = 7.7 Hz, 2H), 6.96 (t, *J* = 7.5 Hz, 2H), 6.81 (d, *J* = 8.1 Hz, 2H), 6.55 (m, 4H), 5.78 (t, *J* = 5.0, 2H), 5.74 (t, *J* = 5.0 Hz, 2H). Diastereomer **11**(η^6-**U**)(η^6-**D**): δ 7.41 (t, *J* = 7.6 Hz, 2H), 7.19 (d, *J* = 7.8 Hz, 2H), 6.96 (m, 4H), 6.58 (d, *J* = 5.1 Hz, 2H), 6.43 (d, *J* = 5.2 Hz, 2H), 5.88 (t, *J* = 5.1, 2H), 5.73(t, *J* = 5.0 Hz, 2H). ^{13}C NMR (100 MHz, CD_3CN) (diastereomer **11**(η^6-**U**)$_2$): δ 128.5, 123.5, 121.7, 121.3, 111.9, 111.8, 81.3, 76.3, 71.8, 68.5, 58.9 (diastereomer **11**(η^6-**U**)(η^6-**D**)): δ 128.6, 123.8, 121.5, 121.3, 112.5, 112.1, 81.2, 76.1, 74.5, 66.6, 60.1. HRMS (ESI+) *m/z* calcd. for $C_{24}H_{18}N_2Re$ [M]$^+$: 521.1022, found: 521.1025 (A), 521.1012 (B).

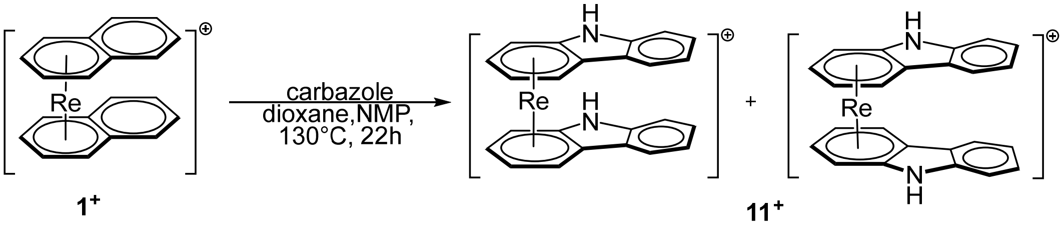

Scheme S4. Synthesis of [Re(η^6-carbazole)$_2$]$^+$ (**11$^+$**).

Synthesis of [Re(η^6-melatonin)$_2$]TFA (13)TFA: Solution A: (**1**)PF$_6$ (30g, 0.05mmol, 1eq.) was dissolved in dry NMP (250μL). The red solution was put in a glass syringe (250μL, Hamilton), which was attached to an automatic pump. Solution B: Two neck Schlenk tube was charged with melatonin (292mg, 1.25mmol, 25eq.) and dry NMP (0.3mL) under N_2, and heated to 120°C, to form clear solution. Then solution A was added dropwise with flow rate of 30μL/h. The addition of solution A was completed in 10h; the reaction was further heated at 120°C for other 11h under N_2. The solvent was removed under vacuum and the brown crude was washed with diethyl ether (1.5mL x 4), three times with a mixture of acetonitrile/diethyl ether (0.2mL/8mL), and then with dry diethyl ether (8mL). The brown crude was purified by preparative-HPLC/**G7**, obtaining three

fractions of (**13**)TFA: pure diastereomer A, B and a mixture of the two. The three fractions were freeze-dried to afford A = 3mg and B = 2mg and A + B = 9mg as yellow powder (total yield: 34%). Overall integration of ^{1}H NMR of three fractions resulted 57% A and 43% B. ^{1}H NMR (500 MHz, CD_3OD) (isomer A): δ 7.27 (s, 2H), 6.68 (s, 2H), 6.09 (d, *J* = 5.4 Hz, 2H), 5.80 (q, *J* = 2.1 Hz, 2H), 3.64 (s, 6H), 3.38 (m, *J* = 3.4 Hz, 4H), 2.64 (t, *J* = 7.5Hz, 4H), 1.95 (s, 6H); (isomer B): δ 7.15 (s, 2H), 6.67 (s, 2H), 6.17 (d, *J* = 5.4 Hz, 2H), 5.88 (q, *J* = 2.1 Hz, 2H), 3.58 (s, 6H), 3.39 (t, *J* = 7.5 Hz, 4H), 2.65 (m, *J* = 3.7 Hz, 4H), 1.95 (s, 6H). ^{13}C NMR (125 MHz, CD_3CN) (isomer A): δ 173.4, 131.2, 130.8, 117.6, 107.0, 87.0, 64.2, 57.6, 55.8, 54.8, 40.5, 26.1, 22.7; (isomer B): δ 173.5, 130.9, 130.0, 116.7, 106.5, 86.3, 63.9, 57.3, 56.1, 53.8, 40.5, 25.9, 22.6. HRMS (ESI+) *m/z* calcd. for $C_{26}H_{32}N_4O_4Re$ $[M]^+$: 651.1976, found: isomer A= 651.1969, isomer B=651.1967.

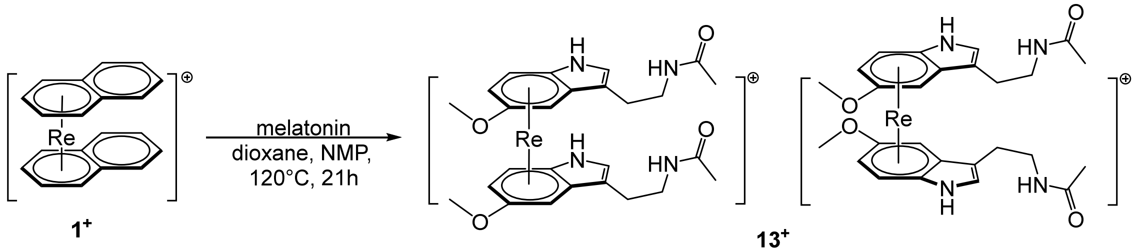

1$^+$ **13**$^+$

Scheme S5. Synthesis of $[Re(\eta^6\text{-melatonin})_2]^+$ (**13**$^+$).

Synthesis of [Re(η^6-mequinol)(η^6-naphthalene)](PF_6) (14)(PF_6): (**1**)PF_6 (30mg, 0.051mmol, 1eq.) was mixed with 4-methoxyphenol (316mg, 2.55mmol, 50eq.) and dry NMP (49.20µL, 0.51mmol, 10eq.) in a Schlenk tube, which was heated at 130°C under N_2 for 14h. The black residue was washed with Et_2O (3x2.5mL), purified by preparative HPLC/**G4** gradient and freeze-dried. After the addition of NH_4PF_6 (10mg, 0.065mmol), the product (**14**)PF_6 was isolated as a pale yellow solid: 13mg (44%). Single crystals, suitable for X-ray diffraction analysis, were obtained by vapor diffusion from Et_2O (anti solvent) into methanol (solvent). ^{1}H NMR (400 MHz, acetone-*d$_6$*) δ 7.62 (m, 2H,), 7.44 (dd, *J* = 6.8, 3.2 Hz, 2H), 6.84 (m, 2H), 6.06 (m, *J* = 7.4, 4.9 Hz, 4H), 5.95 (d, *J* = 5.5 Hz, 2H), 3.48 (s, 3H) ^{13}C NMR (100 MHz, acetone-*d$_6$*) δ 131.6, 131.5, 93.4, 80.4, 71.2, 64.9, 64.1, 57.5. HRMS (ESI$^+$) *m/z* calcd. for $C_{17}H_{16}O_2Re$ $[M]^+$: 439.0702, found: 439.0697.

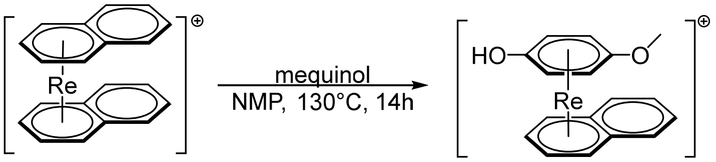

1$^+$ **14**$^+$

Scheme S6. Synthesis of $[Re(\eta^6\text{-mequinol})(\eta^6\text{-naphthalene})]^+$(**14**$^+$).

Synthesis of [Re(η^6-benzene)(η^6-hexestrol)]TFA (17)TFA: (**15**)PF_6 (20mg, 0.037mmol, 1eq.) was partially dissolved in 2mL of 1,4-dioxane and mixed with hexestrol (125.0mg, 0.47mmol, 25eq.) and dry NMP (9µL, 0.093mmol, 5eq.) in a Schlenk tube, which was heated at 120°C under N_2 for 14h. The solvent was removed under vacuum and the yellow residue was purified by preparative HPLC/**G8** gradient and freeze-dried to afford (**17**)TFA (3mg, 12%) as a pale-yellow solid. Single crystals, suitable for X-ray diffraction, analysis were obtained by slowly evaporation of a methanol solution of (**17**)TFA. ^{1}H NMR (400 MHz, acetone-*d$_6$*) δ 6.89 (m, 2H), 6.78 (m, 2H), 6.44 (dd, *J* = 5.9, 1.5 Hz, 1H), 6.23 (dd, *J* = 5.7, 1.5 Hz, 1H), 5.96 (dd, *J* = 5.9, 1.2 Hz, 1H), 5.75 (s, 6H), 5.38 (dd, *J* = 5.8, 1.2 Hz, 1H), 3.05 (s, 43H), 2.82 (dt, *J* = 12.1, 4.2 Hz, 3H), 2.24 – 2.15 (m, 1H), 1.80 (ddd, *J* = 13.9, 9.5, 7.2 Hz, 1H), 1.70 (dqd, *J* = 14.6, 7.5, 3.6 Hz, 1H), 1.50 (ddt, *J* = 19.0, 14.1, 7.1 Hz, 1H), 1.34 (dqd, *J* = 15.1, 7.6, 3.2 Hz, 1H), 0.95 (t, *J* = 7.4 Hz, 3H), 0.72 (t, *J* =

7.2 Hz, 3H).^{13}C NMR (100 MHz, acetone-d_6) δ 156.9, 133.3, 130.6, 115.8, 103.6, 79.0, 76.5, 76.0, 69.3, 68.6, 52.3, 50.3, 26.3, 22.4, 13.2, 12.6 HRMS (ESI$^+$) *m/z* calcd. for $C_{24}H_{28}O_2Re$ $[M]^+$: 535.1641, found: 535.1645.

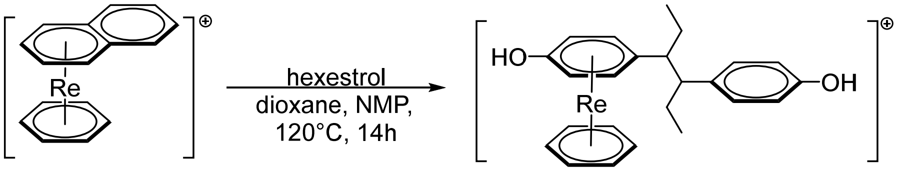

15⁺ **17⁺**

Scheme S7. Synthesis of [Re(η[6]-hexestrol)(η[6]-naphthalene)]$^+$(**17$^+$**).

Synthesis of [Re(η^6-benzene)(η^6-mequinol)]TFA (18)TFA: (**15**)PF_6 (30mg, 0.056mmol, 1eq.) was mixed with 4-methoxyphenol (346.0mg, 2.79mmol, 50eq.) and dry NMP (27μL, 0.279mmol, 5eq.) in a Schlenk tube, which was heated at 130°C under N_2 for 15h. The solvent was removed under vacuum; the black residue was washed with Et_2O/heptane 2/1 (3mL x 3) to obtain a black powder that was purified by preparative HPLC/**G9** gradient and freeze-dried to afford (**18**)TFA (15mg, 52%) as a pale yellow solid. Single crystals, suitable for X-ray diffraction analysis, were obtained by vapor diffusion from Et_2O (antisolvent) into acetonitrile (solvent). ^{1}H NMR (400 MHz, CD_3OD) δ 6.35 (m, 2H), 6.24 (m, 2H), 5.76 (s, 6H), 3.57 (s, 3H).^{13}C NMR (100 MHz, CD_3OD) δ 130.9, 129.9, 76.5, 66.9, 66.2, 57.6. HRMS (ESI$^+$) *m/z* calcd. for $C_{13}H_{14}O_2Re$ $[M]^+$: 389.0549, found: 389.5452.

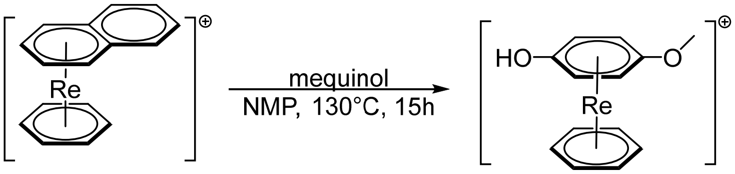

15⁺ **18⁺**

Scheme S8. Synthesis of [Re(η[6]-benzene)(η[6]-mequinol)]$^+$(**18$^+$**).

Synthesis of [Re(η^6-melatonin)(η^6-benzene)]TFA (19)TFA: (**15**)PF_6 (30mg, 0.056mmol, 1eq.) was partially dissolved in 2mL of dry 1,4-dioxane and mixed with melatonin (161.8mg, 0.70mmol, 25eq.) and dry NMP (13.4μL, 0.14mmol, 2.5eq.) in a Schlenk tube, which was heated at 120°C for 3.5h under N_2. The solvent was removed under vacuum and the residue was purified by preparative HPLC/**G10** gradient and freeze-dried to afford (**19**)TFA (23mg, 67%) as a pale yellow solid. ^{1}H NMR (400 MHz, acetone-d_6) δ 10.14 (br s, 1H,), 7.64 (s, 1H), 7.58 (d, 1H), 7.40 (br s, 1H), 7.33 (d, *J* = 5.8 Hz, 1H), 6.30 (d, *J* = 5.8 Hz, 1H), 5.51 (s, 6H) 3.73 (s, 3H), 3.45 (m partially overlapped with H_2O) 2.87 (t, *J* = 7.3 Hz, 2H), 1.90 (s, 3H). ^{13}C NMR (101 MHz, acetone-d_6) δ 133.0, 130.2, 116.4, 107.5, 90.7, 74.5, 67.5, 61.3, 60.6, 57.4, 39.2, 25.5, 22.7. HRMS (ESI$^+$) *m/z* calcd. for $C_{19}H_{22}N_2O_2Re$ $[M]^+$: 497.1233, found: 497.1229.

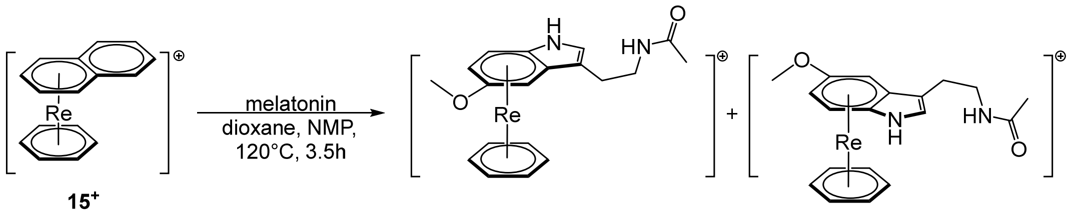

15⁺

19⁺

Scheme S9. Synthesis of [Re(η[6]-benzene)(η[6]-melatonin)]$^+$(**19$^+$**).

General procedure for the syntheses of [^{99m}Tc(η^6-arene)$_2$]$^+$ complexes

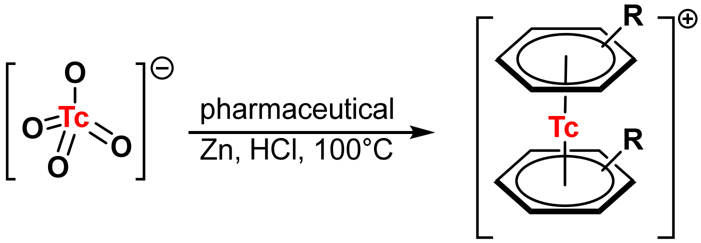

Zn (10-12mg powder or 20-30mg turnings), SDS (4mg), the corresponding arene ligand (0.1 mmol 0.3mmol), 1N or 3N HCl (50μL), were added into the vial. The vial was sealed and flushed with N_2 for 1min. Na[$^{99m}TcO_4$] (1mL) was added, the vial flushed with N_2 for 1 min, and heated at 100°C (μwave) for 35-60min (Table S1 for details). The authenticities of the products were confirmed by co-injection with the corresponding Re analogue, by HPLC UV/vis-detector paired with γ-detector. The separation of the ^{99m}Tc products from excess ligand or residual [$^{99m}TcO_4$]$^-$ was achieved by analytical HPLC. To get clean co-injections, the ^{99m}Tc products were collected and then mixed with the Re homologues.

Table S1. HPLC retention times and co-injections of [M(η^6-pharmaceutical)$_2$]$^+$ (M=^{99m}Tc, Re) complexes **Note:** The ΔR_t values are not constant since the lipo/hydrophilicities between Re and ^{99m}Tc complexes may be slightly different as observed elsewhere.[8]

Entry	Pharmaceuticals	Conditions	Recovery (RCY)[a]	R_t ^{99m}Tc (min)	ΔR_t ^{99m}Tc vs. Re (min)	Figure
1	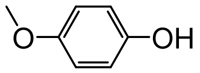	1M HCl<br>Zn turnings<br>60 min	64% (93%)	11.25	0.01	S22
2	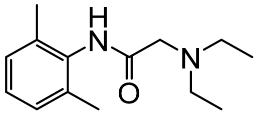	3M HCl<br>Zn powder<br>50 min	13% (92%)	9.15	0.15	S23
3	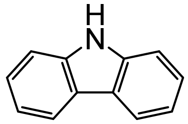	1M HCl<br>Zn turnings<br>35 min	55% (39%)<br>(56%)	18.23<br>18.73	0.02<br>0.14	S24
4	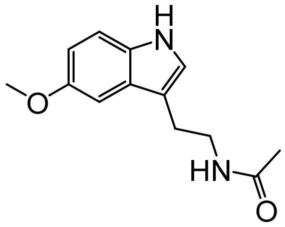	1M HCl<br>Zn powder<br>60 min	15% (71%)[b]	19.80[b]	0.04	S26

a) Determined by radio-HPLC analysis of the crude product. b) The two isomers gave only one trace at the radio-HPLC.

NMR spectra

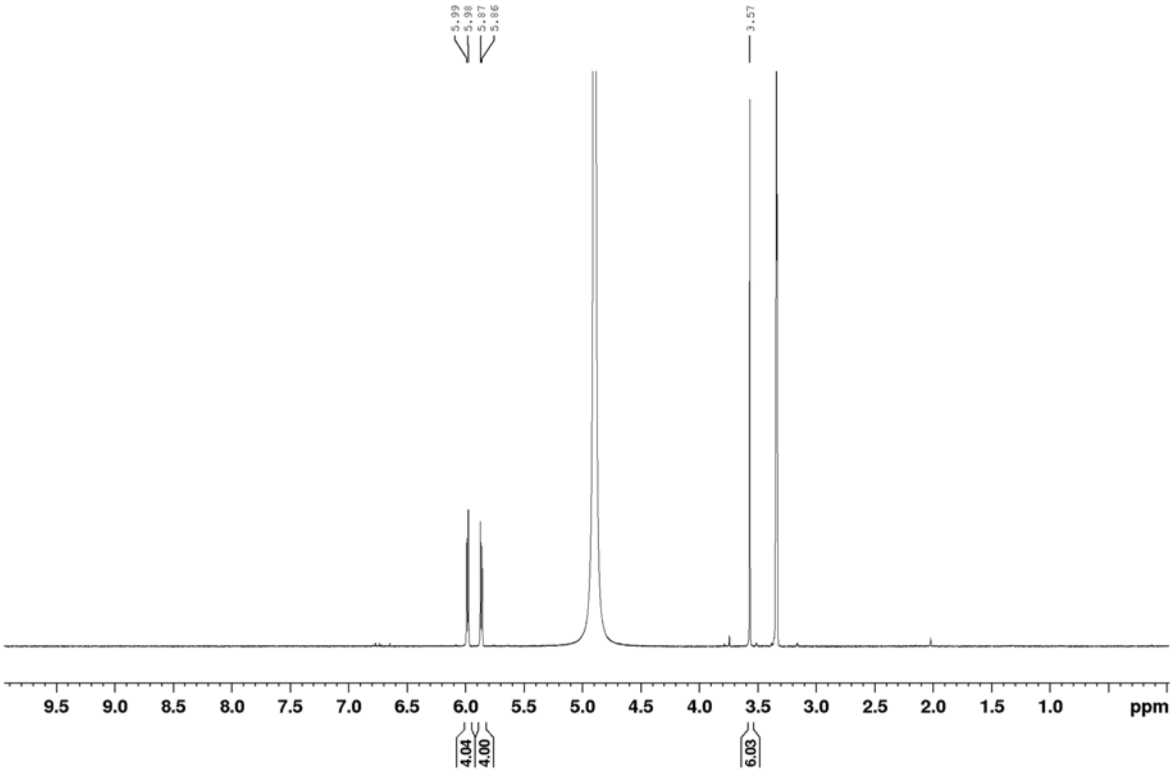

Figure S1.^{1}H NMR spectrum of [Re(η^6-mequinol)$_2$]TFA (**7**)TFA in CD_3OD.

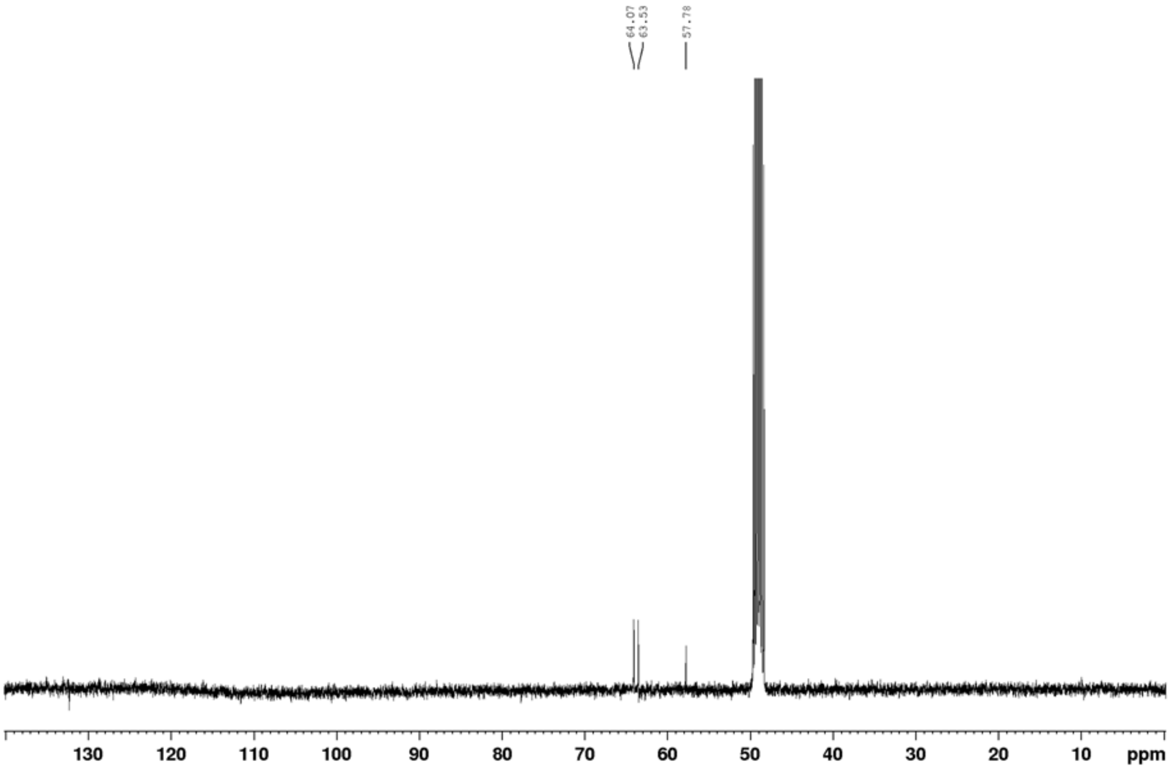

Figure S2.^{13}C NMR spectrum of [Re(η^6-mequinol)$_2$]TFA (**7**)TFA in CD_3OD.

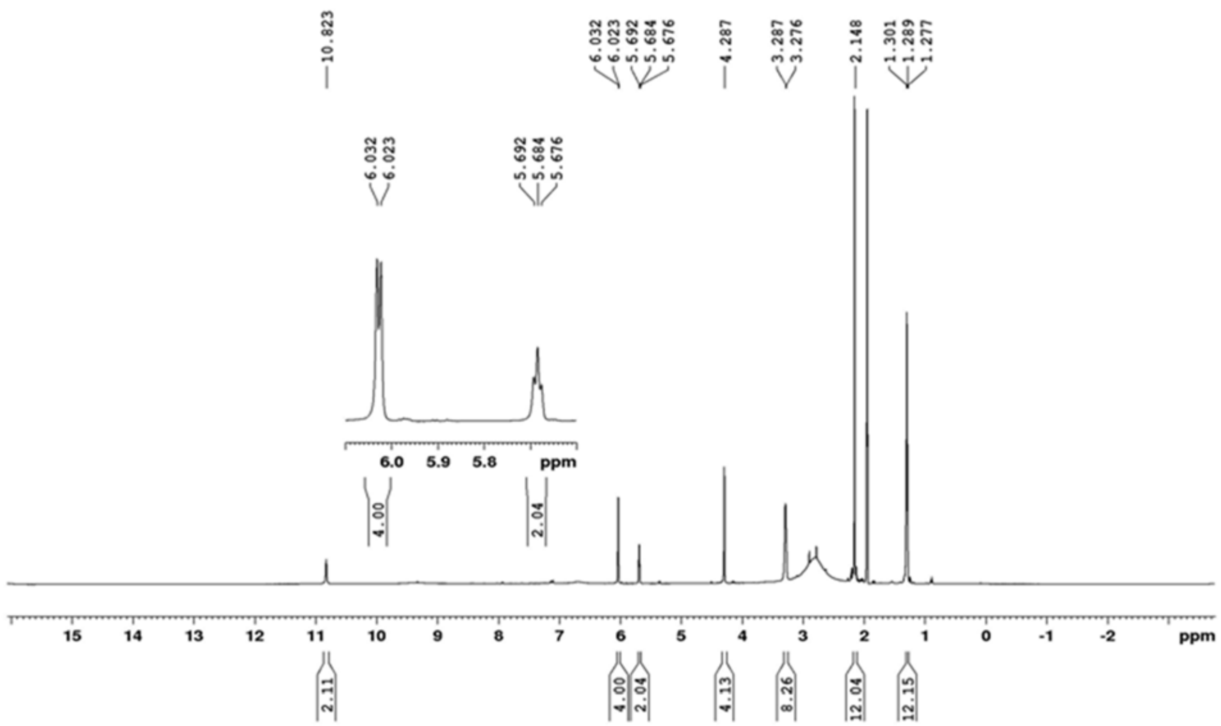

Figure S3.^{1}H NMR spectrum of [Re(η[6]-lidocaine)$_2$]TFA (**9**)TFA in CD_3CN.

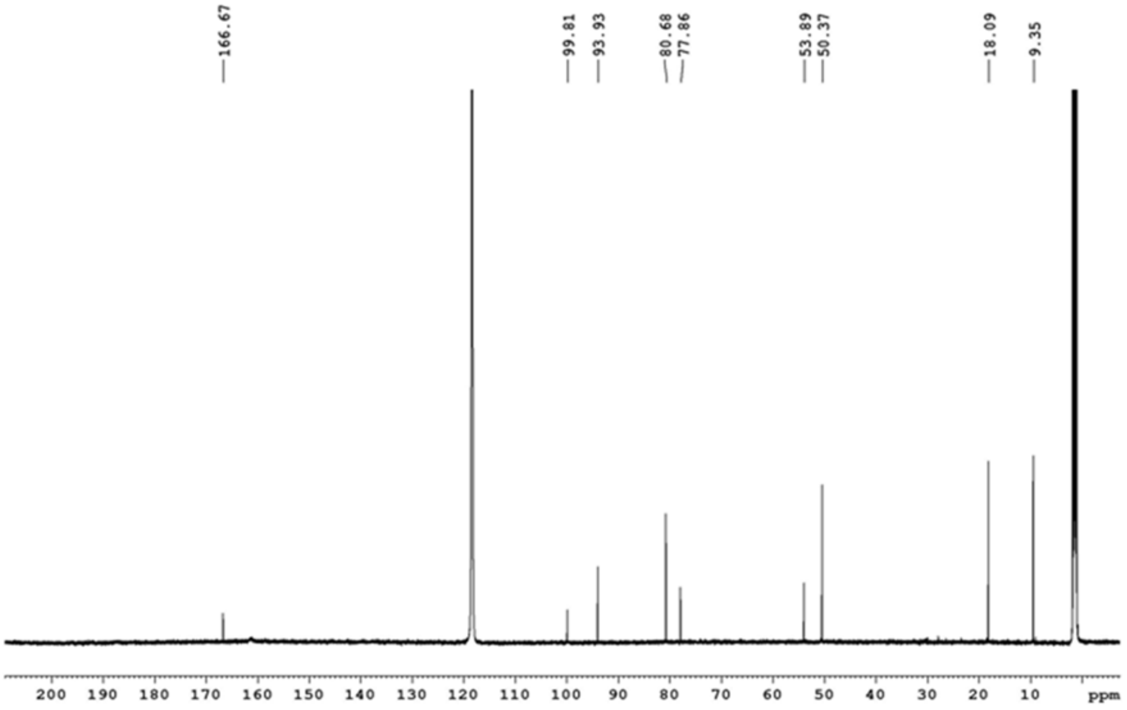

Figure S4.^{13}C NMR spectrum of [Re(η[6]-lidocaine)$_2$]TFA (**9**)TFA in CD_3CN.

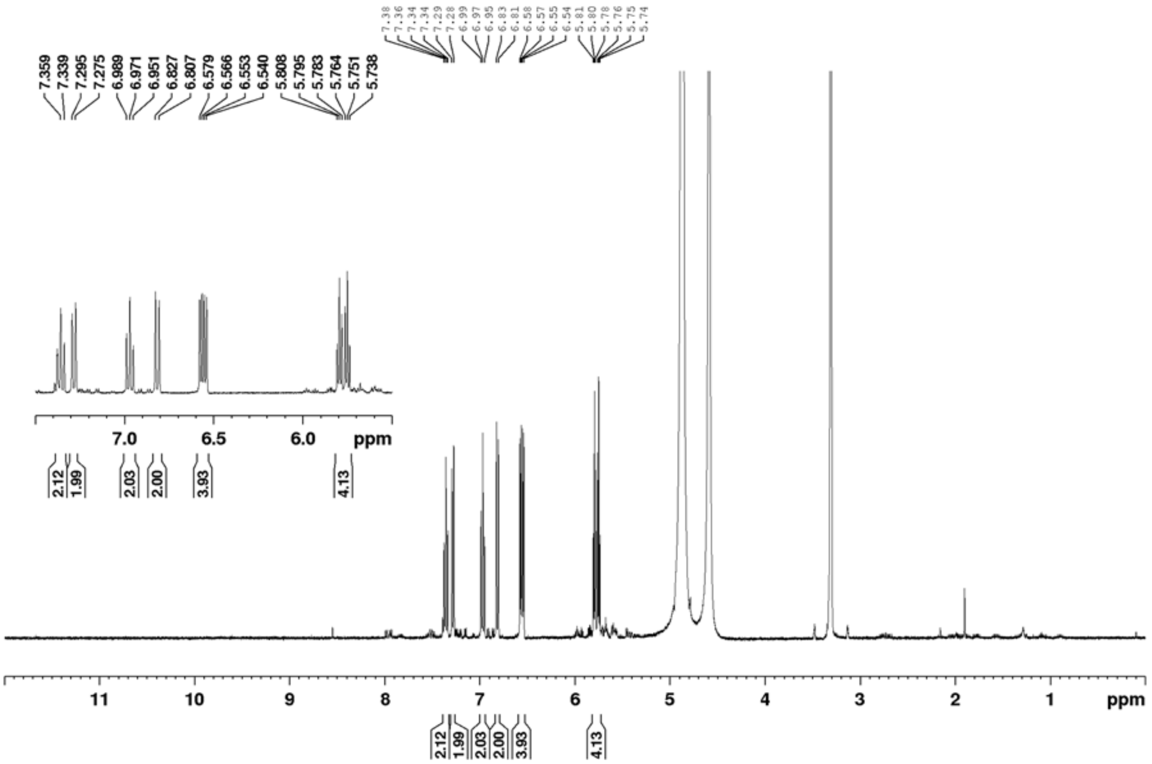

Figure S5.^{1}H NMR spectrum of fraction 1 of [Re(η[6]-carbazole)$_2$]TFA (**11**(η[6]-**U**)$_2$)BF_4 (with some impurities) in CD_3OD.

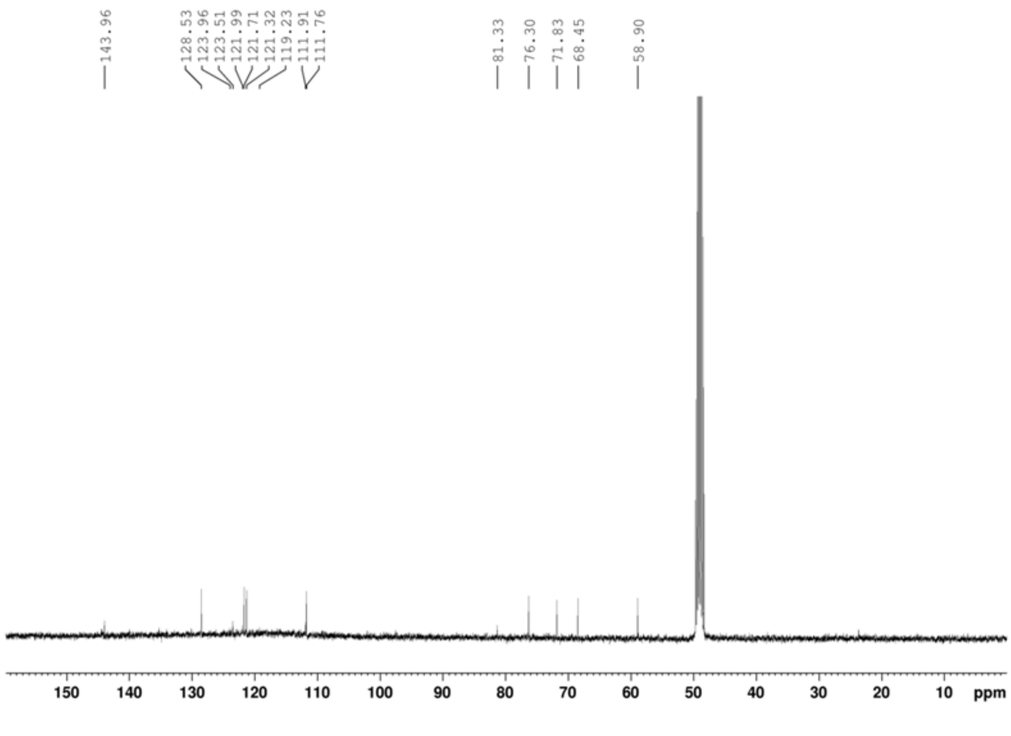

Figure S6.^{13}C NMR spectrum of fraction 1 of [Re(η[6]-carbazole)$_2$]TFA (**11**(η[6]-**U**)$_2$)BF_4 (with some impurities) in CD_3OD.

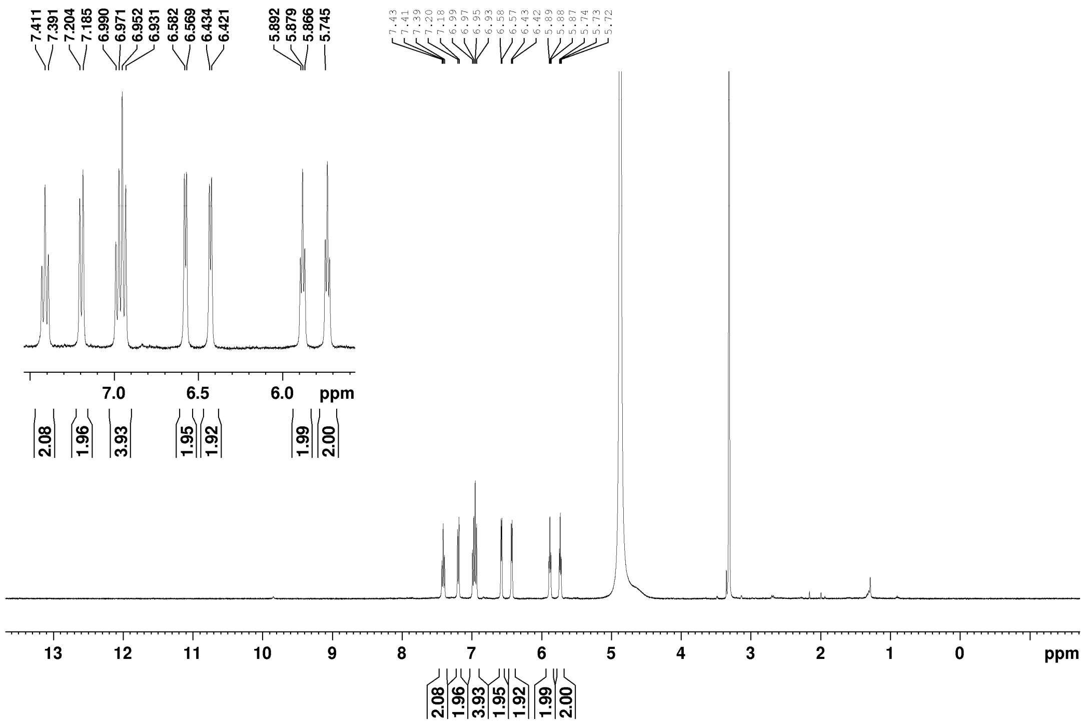

Figure S7.^{1}H NMR spectrum of fraction 2 of [Re(η^6-carbazole)$_2$]TFA (**11**(η^6-**U**)(η^6-**D**))TFA in CD_3OD.

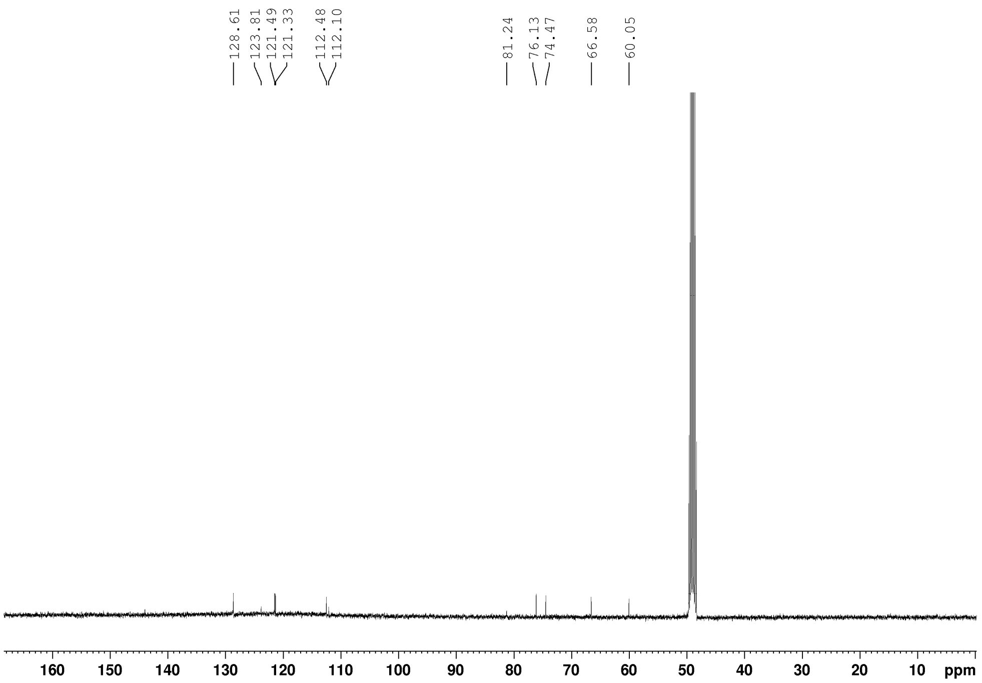

Figure S8.^{13}C NMR spectrum of fraction 2 of [Re(η^6-carbazole)$_2$]TFA (**11**(η^6-**U**)(η^6-**D**))TFA in CD_3OD.

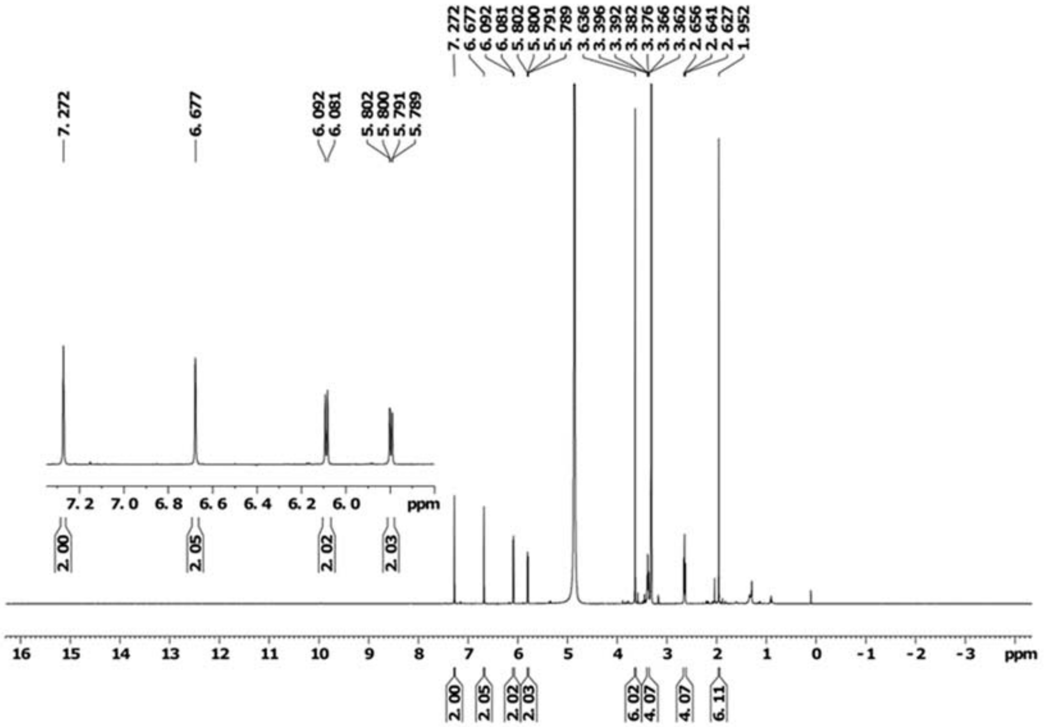

Figure S9.^{1}H NMR spectrum of isomer A [Re(η^{6}-melatonin)$_2$]TFA (**13**)TFA in MeOD.

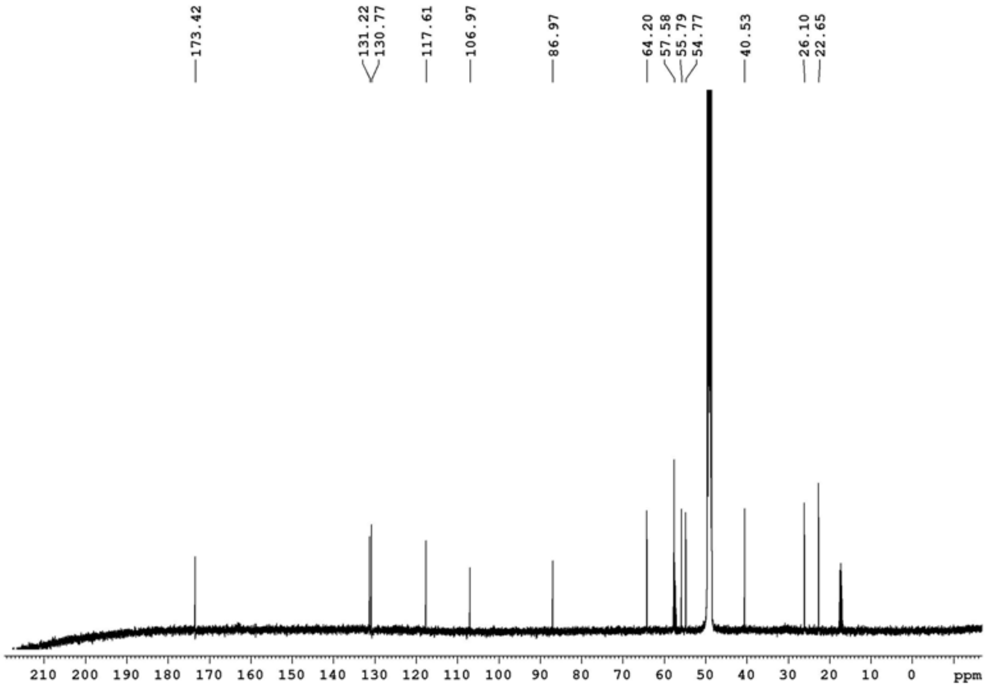

Figure S10.^{13}C NMR spectrum of isomer A [Re(η^{6}-melatonin)$_2$]TFA (**13**)TFA in MeOD.

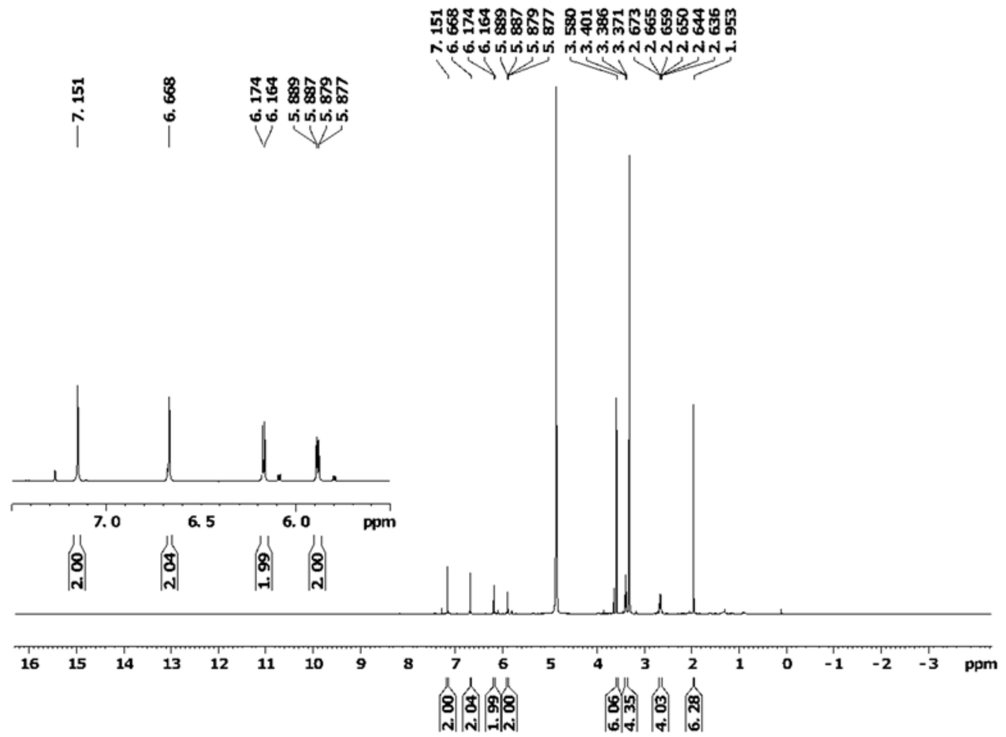

Figure S11.^{1}H NMR spectrum of isomer B [Re(η[6]-melatonin)$_2$]TFA (**13**)TFA in MeOD.

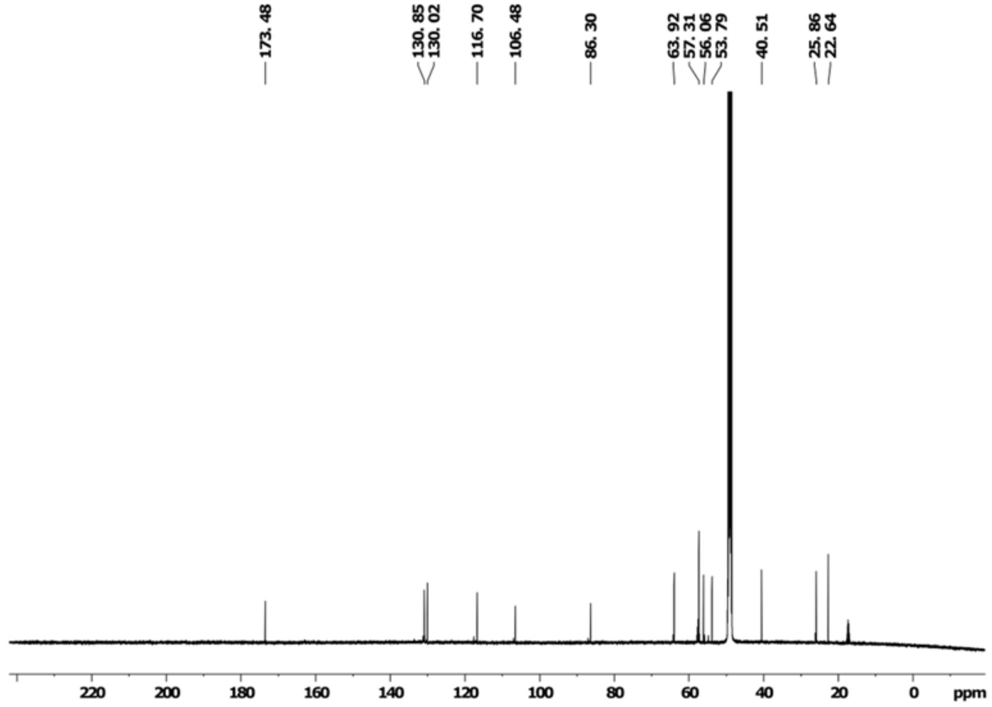

Figure S12.^{13}C NMR spectrum of isomer B [Re(η[6]-melatonin)$_2$]TFA (**13**)TFA in MeOD.

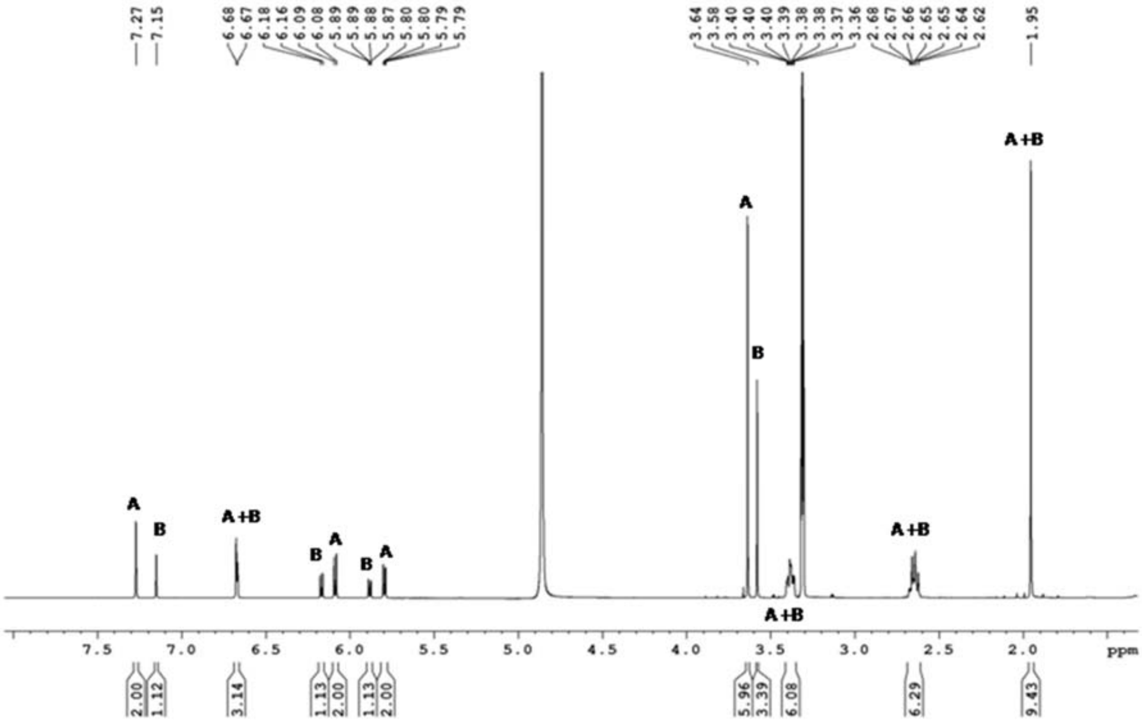

Figure S13.^{1}H NMR spectrum of the mixture of A and B [Re(η^6-melatonin)$_2$]TFA (**13**)TFA in MeOD.

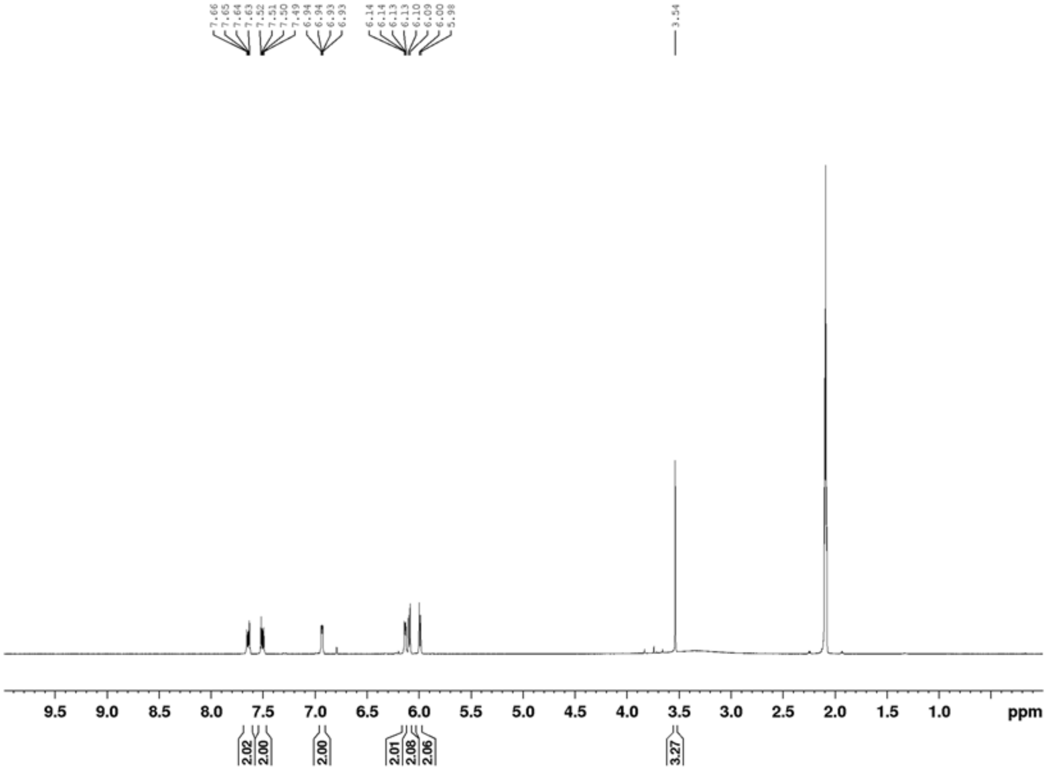

Figure S14.^{1}H NMR spectrum of [Re(η^6-mequinol)(η^6-napht)]PF_6 (**14**)PF_6 in acetone-d_6.

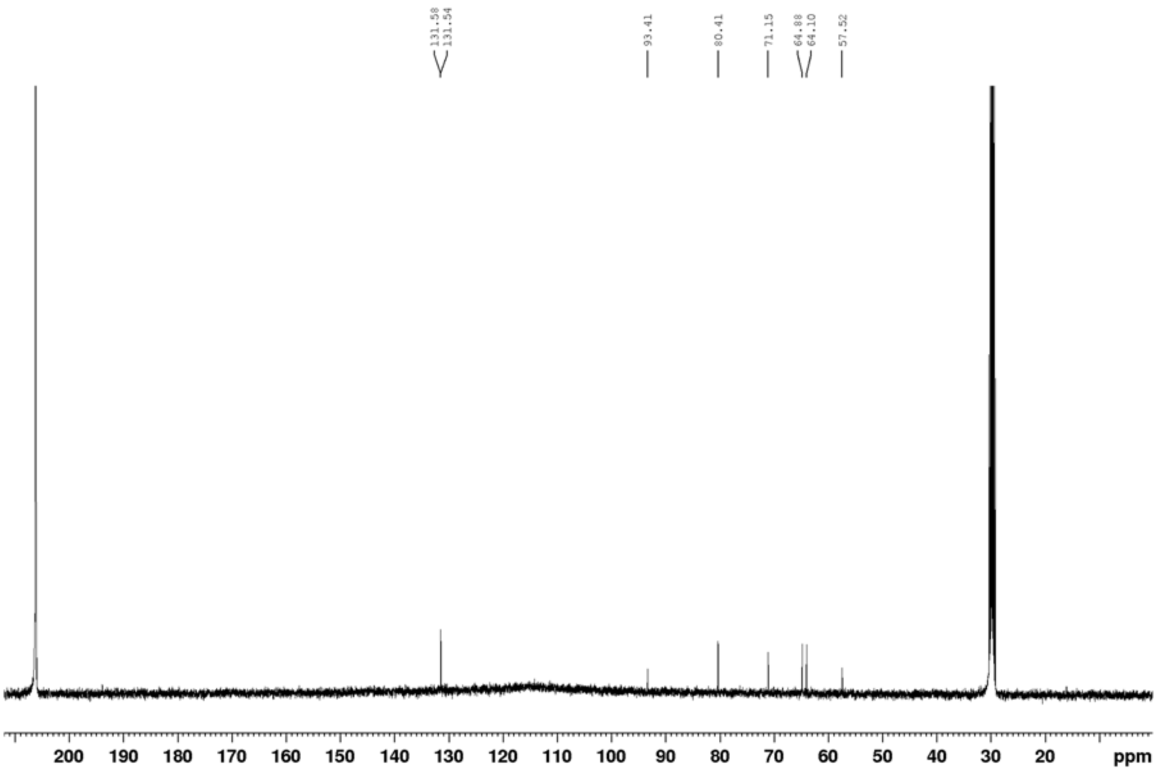

Figure S15. ^{13}C NMR spectrum of [Re(η^6-mequinol)(η^6-napht)]PF_6 (**14**)PF_6 in acetone-d_6.

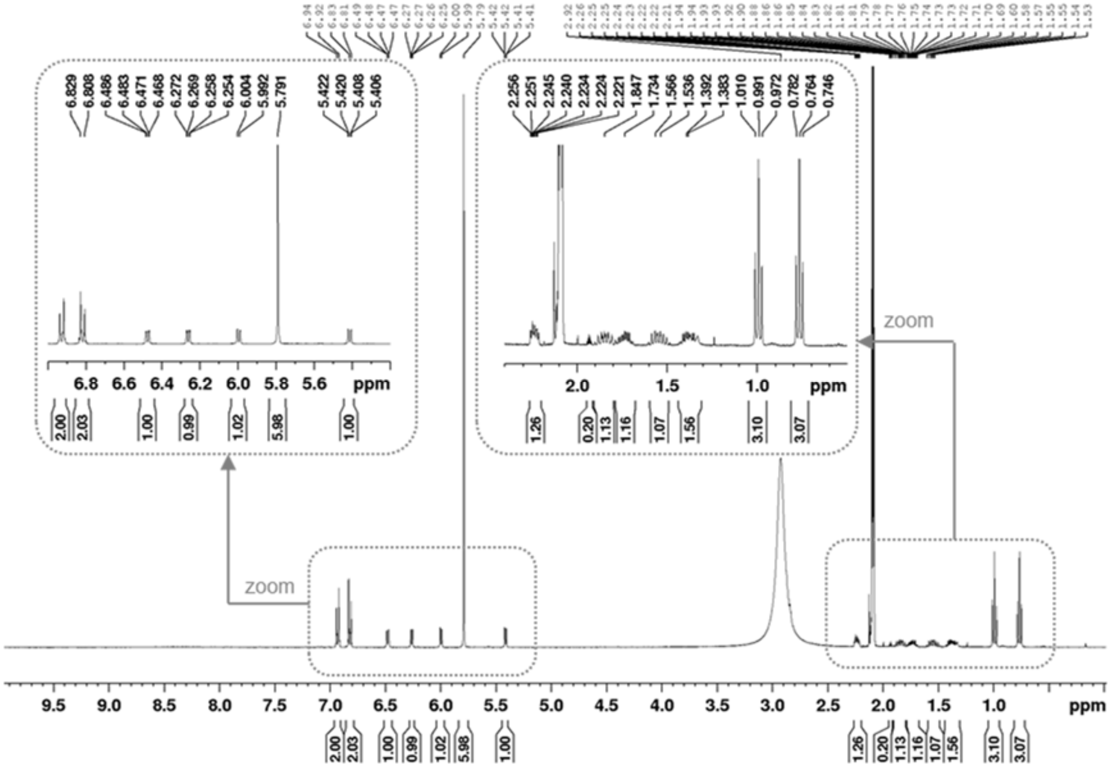

Figure S16. ^1H NMR spectrum of [Re(η^6-C_6H_6)(η^6-hexestrol)]TFA (**17**)TFA in acetone-d_6.

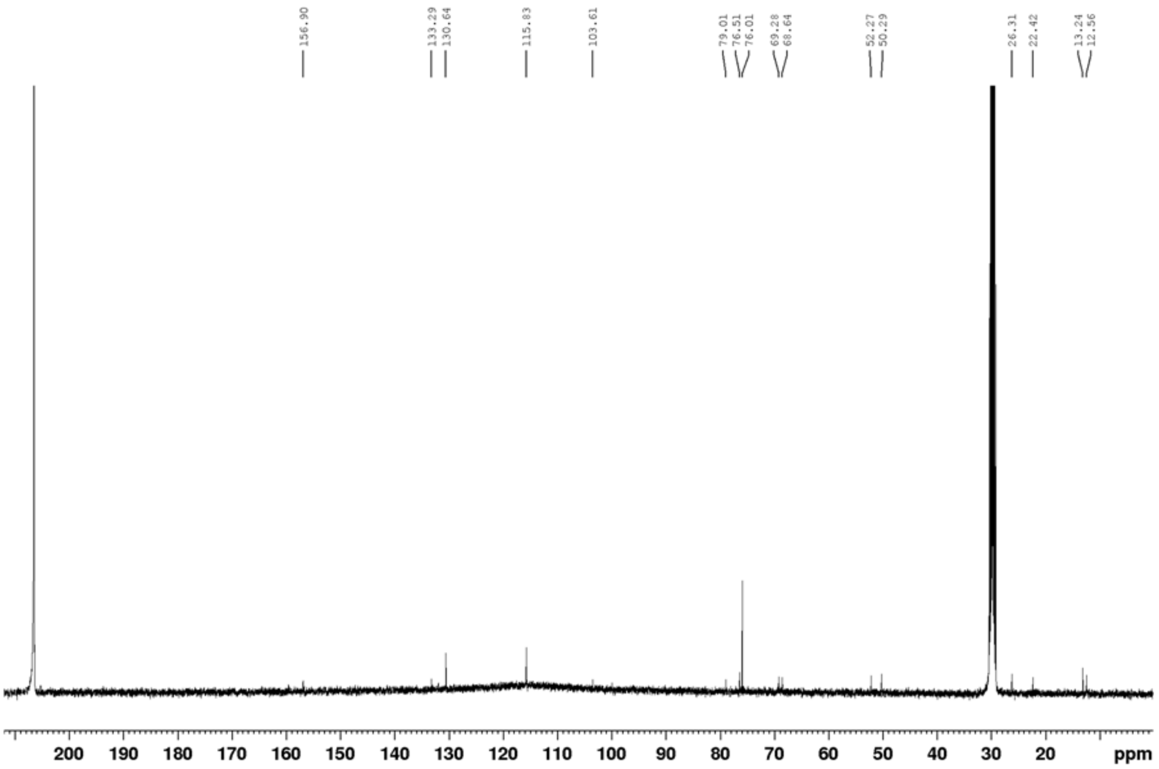

Figure S17.^{13}C NMR spectrum of [Re(η[6]-C_6H_6)(η[6]-hexestrol)]TFA (**17**)TFA in acetone-*d*$_6$.

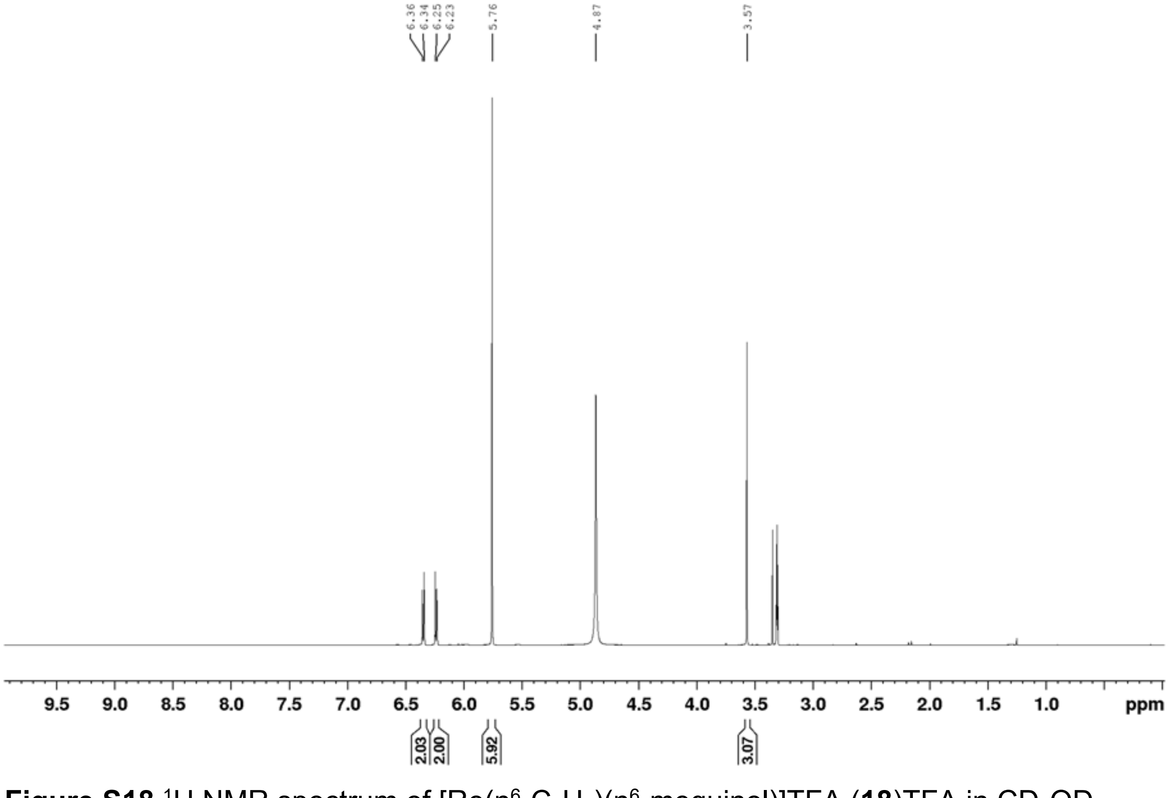

Figure S18.^{1}H NMR spectrum of [Re(η[6]-C_6H_6)(η[6]-mequinol)]TFA (**18**)TFA in CD_3OD.

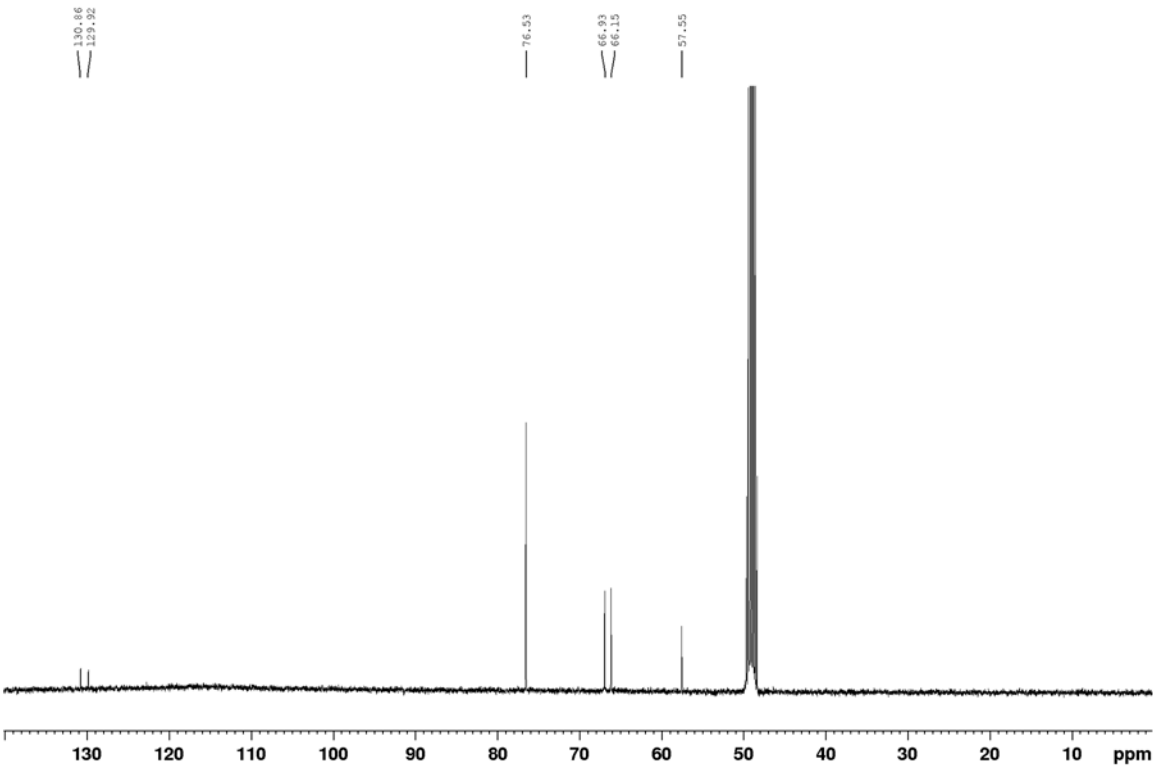

Figure S19.^{13}C NMR spectrum of [Re(η[6]-C_6H_6)(η[6]-mequinol)]TFA (**18**)TFA in CD_3OD.

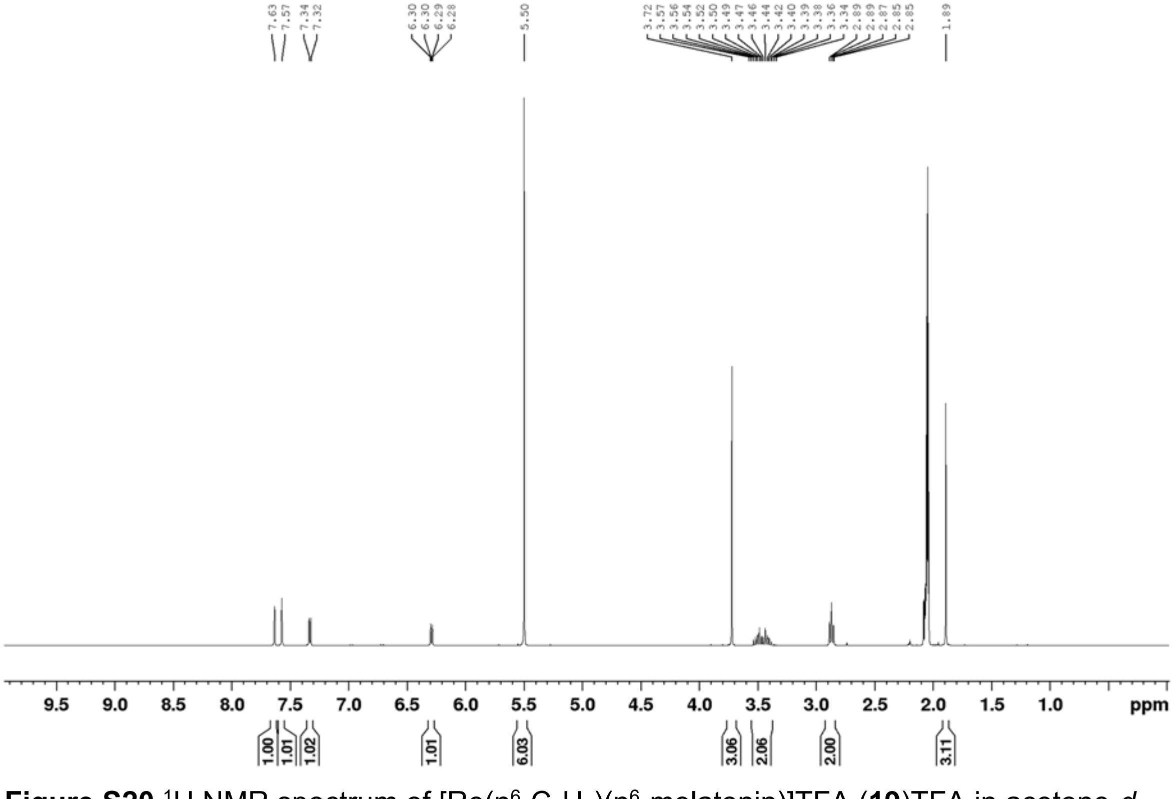

Figure S20.^{1}H NMR spectrum of [Re(η[6]-C_6H_6)(η[6]-melatonin)]TFA (**19**)TFA in acetone-d_6.

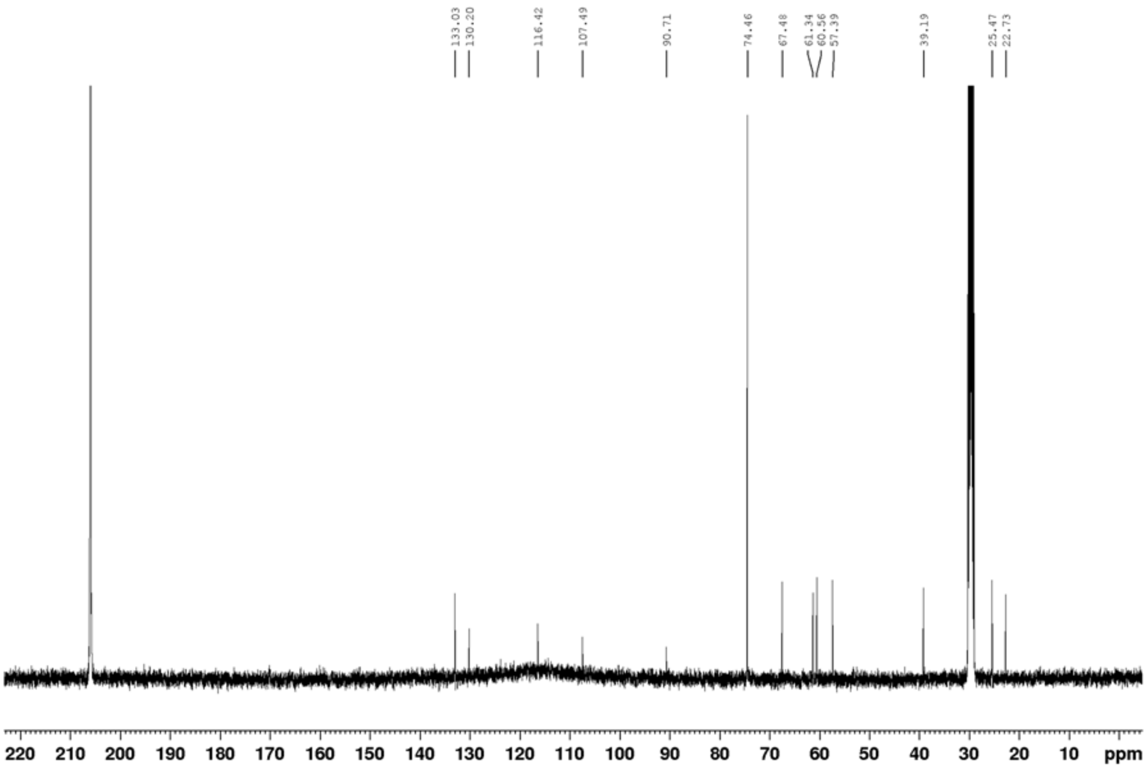

Figure S21.^{13}H NMR spectrum of [Re(η^6-C_6H_6)(η^6-melatonin)]TFA (**19**)TFA in acetone-*d*$_{6.}$

^{99m}Tc labelling

HPLC co-injections

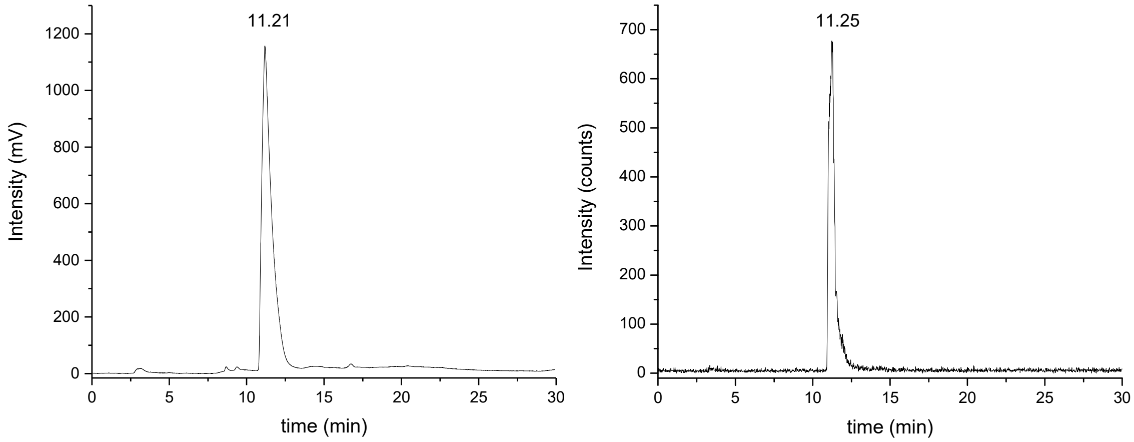

Figure S22: HPLC traces of [Re(η^6-mequinol)$_2$]$^+$ (**7$^+$**) and [^{99m}Tc(η^6-mequinol)$_2$]$^+$.

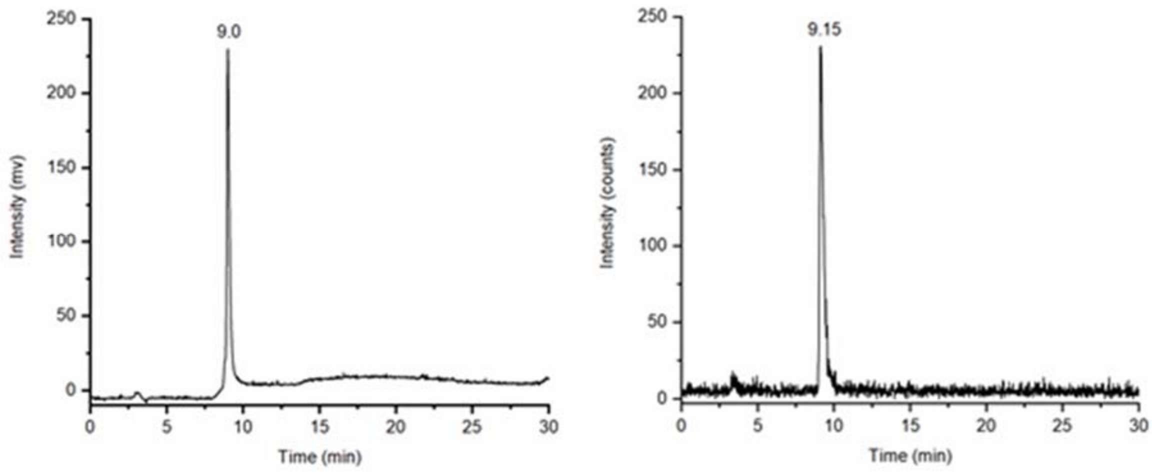

Figure S23: HPLC traces of [Re(η^6-lidocaine)$_2$]$^+$ (**9$^+$**) and [^{99m}Tc(η^6-lidocaine)$_2$]$^+$.

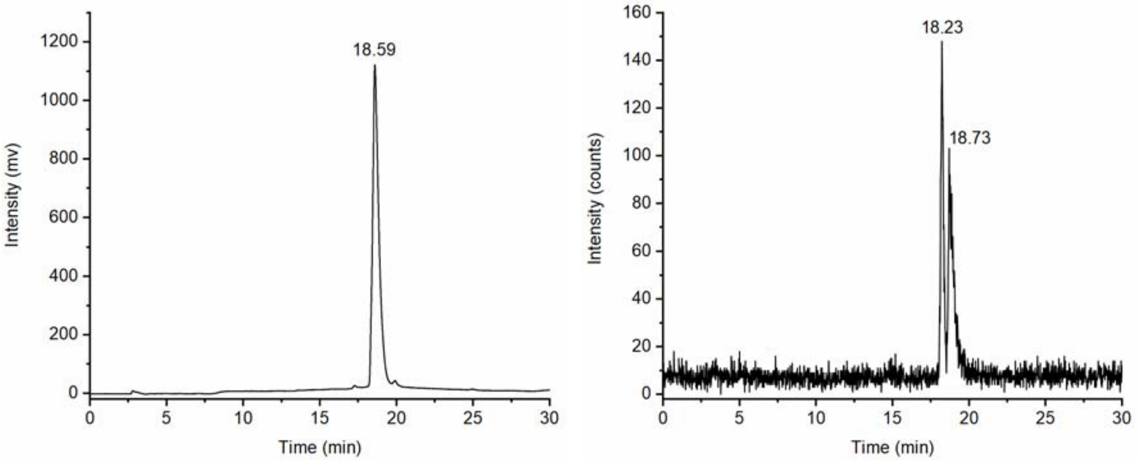

Figure S24: HPLC traces of $[Re(\eta^6\text{-carbazole})_2]^+$(**11**($\eta^6$-**U**)($\eta^6$-**D**)$^+$) and $[^{99m}Tc(\eta^6\text{-carbazole})_2]^+$.

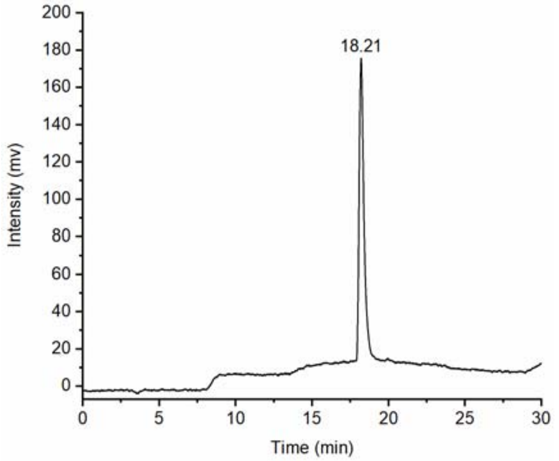

Figure S25: HPLC UV-trace of $[Re(\eta^6\text{-carbazole})_2]^+$ (**11**(η^6-**U**)$_2^+$).

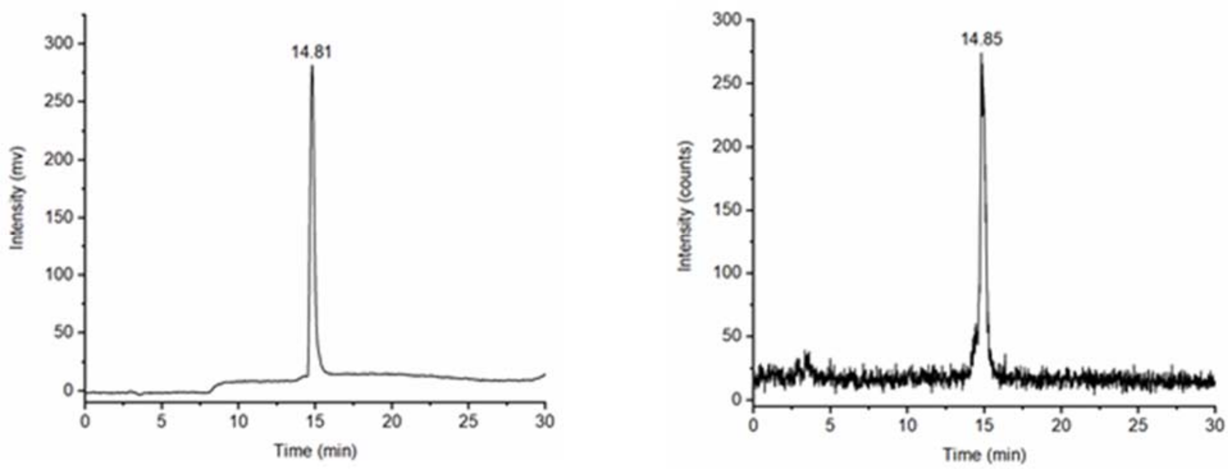

Figure S26: HPLC traces of fraction B of $[Re(\eta^6\text{-melatonin})_2]^+$(**13$^+$**) and $[^{99m}Tc(\eta^6\text{-melatonin})_2]^+$.

HPLC crude γ -traces

All traces are done using **G1** method unless otherwise stated.

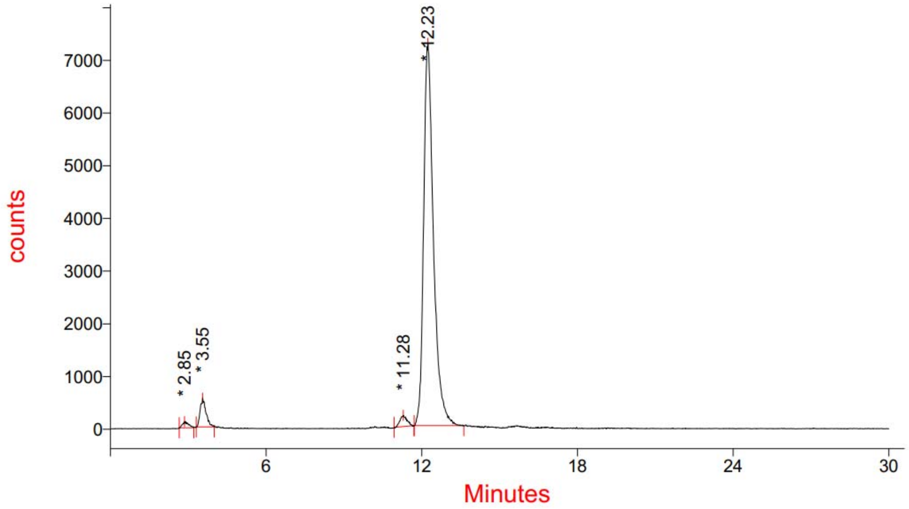

Figure S27. HPLC trace of the crude of [^{99m}Tc(η^6-mequinol)$_2$]$^+$.

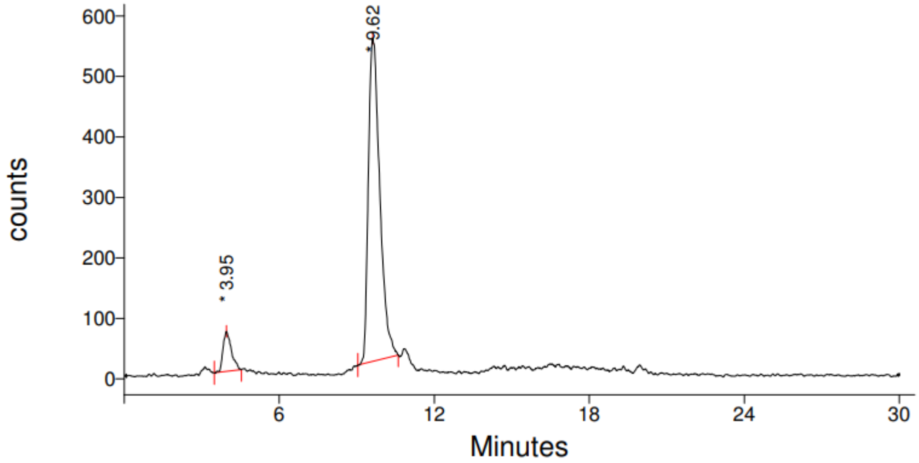

Figure S28. HPLC trace of the crude of [^{99m}Tc(η^6-lidocaine)$_2$]$^+$.

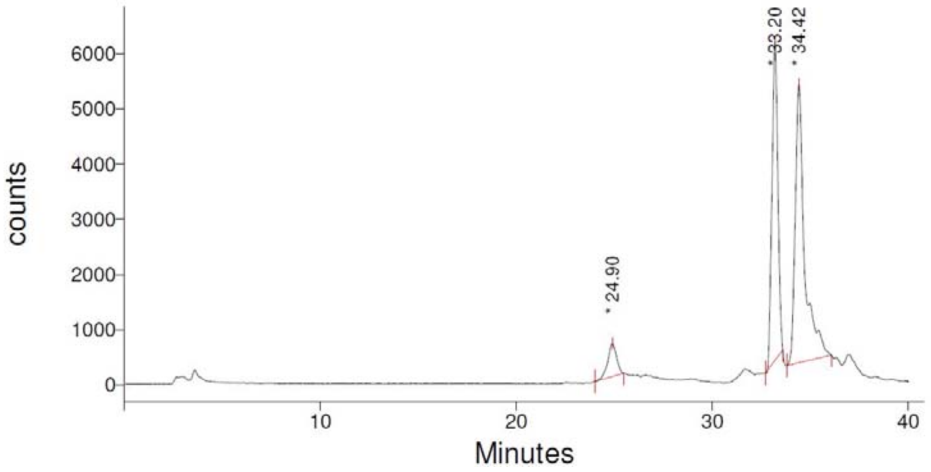

Figure S29. HPLC trace of the crude of [^{99m}Tc(η^6-carbazole)$_2$]$^+$ done using **G2** gradient.

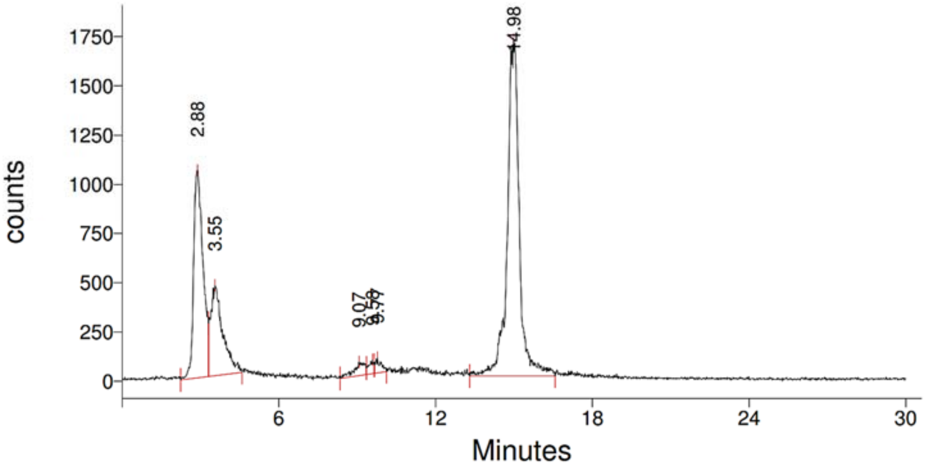

Figure S30. HPLC trace of the crude of [^{99m}Tc(η^6-melatonin)$_2$]$^+$.

X-ray crystallography

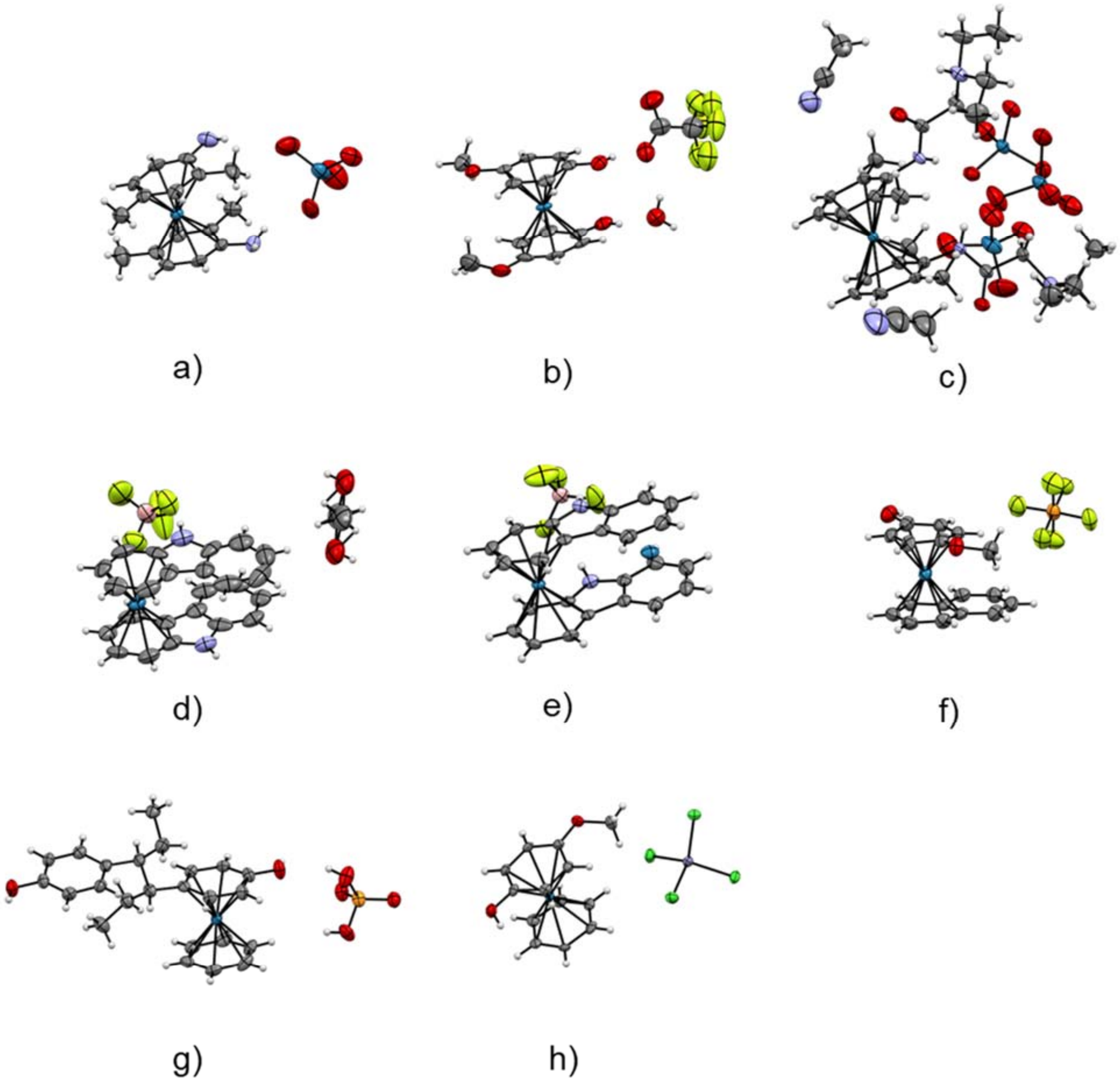

Figure S31. Displacement ellipsoids representation: a) complex [**5**](ReO_4) (showed only one enantiomer) b) complex [**7**](TFA)(H_2O), c) complex [**9**]3(ReO_4)2(CH_3CN), d) complex [**11**](η^6-**U**)$_2$](BF_4)0.25(CH_4O) (showed only one enantiomer), e) complex [**11**](η^6-**U**)(η^6-**D**)]BF_4 (showed only one enantiomer), f) complex [**14**]PF_6, g) complex [**17**](H_2PO_4) (showed only one enantiomer), h) complex([**18**]0.5(Cl_4Zn^2). Thermal ellipsoids represent 50% probability.

Table S2. Crystal data and data collection of complexes [**5**](ReO_4), [**7**](TFA) and [**9**]3(ReO_4)2(CH_3CN).

	**[Re(2,4-dimethylaniline)$_2$](ReO_4)([5](ReO_4)**	**[Re(η^6-mequinol)$_2$](TFA)(H_2O) ([7](TFA)(H_2O))**	**[Re(η^6-lidocaine)$_2$]3(ReO_4^-), 2(CH_3CN)([9]3(ReO_4^-)2(CH_3CN))**
Empirical formula	$C_{16}H_{22}N_2O_4Re_2$	$C_{16}H_{18}F_3O_7Re$	$C_{32}H_{52}N_6O_{14}Re_4$
Formula weight	678.75	565.50	1489.59
Temperature/K	160(1)	160(1)	160(1)
Crystal system	monoclinic	monoclinic	triclinic
Space group	$P2_1/c$	$P2_1/c$	P-1
a/Å	8.02631(6)	14.1855(4)	10.9926(4)
b/Å	10.95811(8)	9.9087(2)	13.2657(2)
c/Å	20.12061(16)	13.8916(3)	16.2581(3)
α/°	90	90	73.809(2)
β/°	91.7420(7)	111.833(3)	71.734(2)
γ/°	90	90	82.707(2)
Volume/Å^3	1768.85(2)	1812.54(8)	2160.04(10)
Z	4	4	2
ρ_{calc}g/cm^3	2.549	2.072	2.290
μ/mm^{-1}	26.552	13.742	21.944
F(000)	1256.0	1088.0	1396.0
Crystal size/mm^3	0.11 × 0.05 × 0.03	0.22 × 0.08 × 0.02	0.07 × 0.04 × 0.03
Radiation	CuKα (λ = 1.54184)	Cu Kα (λ = 1.54184)	Cu Kα (λ = 1.54184)
2Θ range for data collection/°	8.794 to 148.948	6.712 to 149.032	5.918 to 136.502
Index ranges	-10 ≤ h ≤ 9, -13 ≤ k ≤ 13, -25 ≤ l ≤ 24	-17 ≤ h ≤ 17, -12 ≤ k ≤ 9, -17 ≤ l ≤ 17	-13 ≤ h ≤ 13, -15 ≤ k ≤ 15, -19 ≤ l ≤ 17
Reflections collected	28705	18680	41812
Independent reflections	3603 [R_{int} = 0.0262, R_{sigma} = 0.0139]	3716 [R_{int} = 0.0280, R_{sigma} = 0.0153]	7902 [R_{int} = 0.0399, R_{sigma} = 0.0260]
Data/restraints/parameters	3603/0/233	3716/130/285	7902/68/548
Goodness-of-fit on F^2	1.143	1.054	1.026
Final R indexes [I>=2σ (I)]	R_1 = 0.0420, wR_2 = 0.0897	R_1 = 0.0298, wR_2 = 0.0791	R_1 = 0.0299, wR_2 = 0.0781
Final R indexes [all data]	R_1 = 0.0426, wR_2 = 0.0899	R_1 = 0.0315, wR_2 = 0.0807	R_1 = 0.0322, wR_2 = 0.0800
Largest diff. peak/hole / e Å^{-3}	2.11/-3.42	1.38/-0.89	3.21/-3.12
CCDC Nr.	2113179	2113182	2113180

Table S3. Crystal data and data collection of complexes [**11**(η^6-**U**)$_2$], [**11**(η^6-**U**)(η^6-**D**)] and[**14**](PF_6).

	**[Re(η^6-carbazole)$_2$]0.25(CH_4O) ([11(η^6-U)$_2$]$BF_4$0.25CH_4O)**	**[Re(η^6-carbazole)$_2$]0.25(CH_4O) ([11(η^6-U)(η^6-D)]BF_4)**	**[Re(η^6-mequinol)(η^6-naphthalene)](PF_6) ([14]PF_6)**
Empirical formula	$C_{24.25}H_{19}BF_4N_2O_{0.25}Re$	$C_{24}H_{18}BF_4N_2Re$	$C_{17}H_{16}F_6O_2PRe$
Formula weight	615.42	607.41	583.47
Temperature/K	160(1)	160(1)	160(1)
Crystal system	monoclinic	monoclinic	triclinic
Space group	$P2_1/c$	$P2_1/n$	P-1
a/Å	14.7115(3)	10.80750(10)	7.0944(2)
b/Å	10.3023(2)	16.7202(2)	11.1364(3)
c/Å	15.2398(4)	11.67260(10)	11.2980(3)
α/°	90	90	83.329(2)
β/°	115.987(3)	107.7510(10)	87.829(2)
γ/°	90	90	78.687(3)
Volume/$Å^3$	2076.24(9)	2008.86(4)	869.24(4)
Z	4	4	2
ρ_{calc}g/cm^3	1.969	2.008	2.229
μ/mm^{-1}	11.915	12.295	15.235
F(000)	1186.0	1168.0	556.0
Crystal size/mm^3	0.19 × 0.08 × 0.02	0.16 × 0.1 × 0.06	0.11 × 0.04 × 0.01
Radiation	Cu Kα (λ = 1.54184)	Cu Kα (λ = 1.54184)	Cu Kα (λ = 1.54184)
2Θ range for data collection/°	6.684 to 157.82	9.554 to 148.996	7.88 to 158.07
Index ranges	-16 ≤ h ≤ 18, -12 ≤ k ≤ 12, -19 ≤ l ≤ 19	-13 ≤ h ≤ 13, -20 ≤ k ≤ 18, -14 ≤ l ≤ 13	-9 ≤ h ≤ 9, -13 ≤ k ≤ 14, -11 ≤ l ≤ 14
Reflections collected	22458	21064	17570
Independent reflections	4404 [R_{int} = 0.0293, R_{sigma} = 0.0206]	4106 [R_{int} = 0.0226, R_{sigma} = 0.0139]	3658 [R_{int} = 0.0587, R_{sigma} = 0.0320]
Data/restraints/parameters	4404/1/304	4106/38/307	3658/166/286
Goodness-of-fit on F^2	1.086	1.190	1.119
Final R indexes [I>=2σ (I)]	R_1 = 0.0550, wR_2 = 0.1368	R_1 = 0.0229, wR_2 = 0.0529	R_1 = 0.0542, wR_2 = 0.1376
Final R indexes [all data]	R_1 = 0.0568, wR_2 = 0.1378	R_1 = 0.0237, wR_2 = 0.0533	R_1 = 0.0576, wR_2 = 0.1417
Largest diff. peak/hole / e Å^{-3}	2.46/-2.23	0.78/-0.87	3.31/-2.19
CCDC Nr.	2113178	2113177	2113183

Table S4. Crystal data and data collection of complexes [**17**](H_2PO_4) and [**18**](Cl_4Zn^2).

	**[Re(η^6-C_6H_6)(η^6-16)](H_2PO_4) ([17] H_2PO_4))**	**[Re(η^6-C_6H_6)(η^6-6)]0.5(Cl_4Zn^2) ([18]Cl_4Zn^{2-})**
Empirical formula	$C_{24}H_{30}O_6PRe$	$C_{26}H_{28}Cl_4O_4Re_2Zn$
Formula weight	631.65	984.05
Temperature/K	160(1)	160(1)
Crystal system	monoclinic	monoclinic
Space group	$P2_1/n$	C2/c
a/Å	12.7589(3)	18.6322(3)
b/Å	14.6623(3)	7.58720(10)
c/Å	13.2006(4)	21.0603(3)
α/°	90	90
β/°	111.803(3)	110.036(2)
γ/°	90	90
Volume/Å^3	2292.84(12)	2797.03(8)
Z	4	4
ρ_{calc}g/cm^3	1.830	2.337
μ/mm^{-1}	11.354	21.340
F(000)	1248.0	1856.0
Crystal size/mm^3	0.19 × 0.15 × 0.08	0.24 × 0.1 × 0.07
Radiation	Cu Kα (λ = 1.54184)	Cu Kα (λ = 1.54184)
2Θ range for data collection/°	8.232 to 148.998	8.938 to 148.978
Index ranges	-15 ≤ h ≤ 15, -14 ≤ k ≤ 18, -15 ≤ l ≤ 16	-23 ≤ h ≤ 23, -6 ≤ k ≤ 9, -26 ≤ l ≤ 26
Reflections collected	22835	13093
Independent reflections	4636 [R_{int} = 0.0316, R_{sigma} = 0.0202]	2858 [R_{int} = 0.0273, R_{sigma} = 0.0148]
Data/restraints/parameters	4636/0/301	2858/396/229
Goodness-of-fit on F^2	1.077	1.186
Final R indexes [I>=2σ (I)]	R_1 = 0.0340, wR_2 = 0.0995	R_1 = 0.0185, wR_2 = 0.0478
Final R indexes [all data]	R_1 = 0.0361, wR_2 = 0.1014	R_1 = 0.0187, wR_2 = 0.0479
Largest diff. peak/hole / e Å^{-3}	1.68/-1.19	0.65/-0.80
CCDC Nr.	2113184	2113181

References:

(1) Clark, R. C.; Reid, J. S. *Acta Cryst. A* **1995**, 51, 887-897.

(2) *CrysAlisPro* (version 1.171.40.68a), Rigaku Oxford Diffraction Ltd, Yarnton, Oxfordshire, England, **2019**.

(3) O.V. Dolomanov, L. J. Bourhis, R. J. Gildea, J. A. K. Howard, H. Puschmann, *J. Appl. Cryst.* **2009**, *42*, 339-341.

(4) G. M. Sheldrick, *Acta Cryst. A* **2015**, *71*, 3-8.

(5) G. M. Sheldrick, *Acta Cryst. C* **2015**, *71*, 3-8.

(6) G. M. Sheldrick, *Acta Cryst. D* **2009**, *65*, 148-155.

(7) Q. Nadeem, G. Meola, H. Braband, R. Bolliger, O. Blacque, D. Hernandez-Valdes, R. Alberto, *Angew. Chem. Int. Ed.* **2020**, *59*, 1197-1200.

(8) M. Benz, H. Braband, P. Schmutz, J. Halter, R. Alberto, *Chem. Sci.* **2015**, *6*, 165-169.
